# Multiscale Model of CVD Growth of Graphene on Cu(111) Surface

**DOI:** 10.3390/ijms24108563

**Published:** 2023-05-10

**Authors:** Meysam Esmaeilpour, Patrick Bügel, Karin Fink, Felix Studt, Wolfgang Wenzel, Mariana Kozlowska

**Affiliations:** 1 Institute of Nanotechnology (INT), Karlsruhe Institute of Technology, Hermann-von-Helmholtz-Platz 1, 76344 Eggenstein-Leopoldshafen, Germany; 2 Institute of Catalysis Research and Technology (IKFT), Karlsruhe Institute of Technology, Hermann-von-Helmholtz-Platz 1, 76344 Eggenstein-Leopoldshafen, Germany; 3 Institute for Chemical Technology and Polymer Chemistry (ITCP), Karlsruhe Institute of Technology, 76131 Karlsruhe, Germany

**Keywords:** CVD growth, graphene, kinetic Monte Carlo, density functional theory, multiscale modeling

## Abstract

Due to its outstanding properties, graphene has emerged as one of the most promising 2D materials in a large variety of research fields. Among the available fabrication protocols, chemical vapor deposition (CVD) enables the production of high quality single-layered large area graphene. To better understand the kinetics of CVD graphene growth, multiscale modeling approaches are sought after. Although a variety of models have been developed to study the growth mechanism, prior studies are either limited to very small systems, are forced to simplify the model to eliminate the fast process, or they simplify reactions. While it is possible to rationalize these approximations, it is important to note that they have non-trivial consequences on the overall growth of graphene. Therefore, a comprehensive understanding of the kinetics of graphene growth in CVD remains a challenge. Here, we introduce a kinetic Monte Carlo protocol that permits, for the first time, the representation of relevant reactions on the atomic scale, without additional approximations, while still reaching very long time and length scales of the simulation of graphene growth. The quantum-mechanics-based multiscale model, which links kinetic Monte Carlo growth processes with the rates of occurring chemical reactions, calculated from first principles makes it possible to investigate the contributions of the most important species in graphene growth. It permits the proper investigation of the role of carbon and its dimer in the growth process, thus indicating the carbon dimer to be the dominant species. The consideration of hydrogenation and dehydrogenation reactions enables us to correlate the quality of the material grown within the CVD control parameters and to demonstrate an important role of these reactions in the quality of the grown graphene in terms of its surface roughness, hydrogenation sites, and vacancy defects. The model developed is capable of providing additional insights to control the graphene growth mechanism on Cu(111), which may guide further experimental and theoretical developments.

## 1. Introduction

Graphene, as the first isolated two-dimensional (2D) material [1], has attracted enormous attention because of its exceptional mechanical [2], electric [3], thermal [4], and optical [5] properties. These features have made graphene a potential candidate for a wide variety of applications in science and technology, such as field-effect transistors [6,7,8], flexible electronics [9,10], photodetectors [11,12], energy storage [13,14], precise sensors [15,16], DNA sequencing [17,18], drug delivery [19,20], and composite materials [21]. Due to this fact, the development of methods and techniques for its large-scale production has received a lot of interest. Two main strategies for synthesizing graphene are bottom-up and top-down approaches [22,23,24]. Bottom-up approaches involve constructing graphene from smaller units such as atoms, molecules, or clusters [25,26], while top-down approaches involve breaking larger graphene structures into smaller pieces [27,28]. There are various bottom-up approaches used for graphene synthesis. Chemical vapor deposition (CVD) involves the decomposition of a carbon-containing gas with a metal catalyst and is promising for large-scale, high-quality graphene production [29]. Molecular beam epitaxy (MBE) allows for precise control over the thickness and quality of graphene layers by depositing individual carbon atoms onto a substrate [30]. Chemical synthesis uses chemical reactions to synthesize graphene from organic molecules and is cost-effective for high-quality graphene production [31]. Top-down approaches for graphene synthesis include mechanical exfoliation, chemical exfoliation, and plasma etching [27]. Mechanical exfoliation involves peeling off thin layers of graphene from a bulk graphite source using adhesive tape [32]. Chemical exfoliation uses reactions such as Hummer’s method to break down graphite into individual graphene sheets [33]. Plasma etching uses a plasma to selectively etch away graphene layers from a bulk graphite source [34]. The selection of the most suitable approach depends on the intended application and desired properties of the graphene material. Among the various bottom-up approaches, chemical vapor deposition (CVD) is considered the most promising for the large-scale production of high-quality graphene. This is due to its scalability and compatibility with existing semiconductor fabrication techniques [35].

The CVD process is highly dependent on the synthetic parameters, such as the partial pressure of precursors, temperature, substrate surface, and carbon solubility in the metal substrate [29,36,37]. All these parameters affect the quality of the material produced. Temperatures of 1073–1300 K, a low flow rate, and a low partial pressure of CH${}_{4}$ are crucial factors for the growth of single-crystalline monolayer domains [38,39,40,41,42,43]. Copper emerged as the most suitable substrate for the synthesis of single-layer graphene [44]. Moreover, its Cu(111) surface has been shown to result in less polycrystalline graphene material [45,46], therefore, the CVD process on the Cu(111) surface is of high interest in both industry and academia.

The controllable synthesis of large-sized graphene using CVD is still a challenge due to the complex growth process and its sensitivity to growth conditions [47]. A detailed understanding of the kinetic mechanisms of the CVD growth of graphene requires in-depth studies from both the experimental and theoretical perspective [48]. Considering the elementary reactions leading to the formation of graphene, the optimal process parameters are still not clear. In addition, a deeper understanding of the kinetics and rate limiting steps is needed [49]. Alnuaimi et al. [50] reported the effect of growth temperature, pressure, and the CH${}_{4}$ to H${}_{2}$ ratio on the growth of high-quality, large-sized graphene in CVD. The results revealed that high temperature (1060 °C) reduced the multilayer nucleation density by more than 50%, while low chamber pressure and the CH${}_{4}$ to H${}_{2}$ ratio controlled the graphene flake size and quality.

The mechanism of graphene growth in CVD has a multiscale character, i.e., it requires multiscale modeling approaches to properly complement experimental investigations. Numerous theoretical studies, employing ab initio calculations [51], molecular dynamics (MD) [52], and kinetic Monte Carlo (KMC) simulations [53,54,55,56,57], have been reported to model the CVD growth mechanisms of graphene and the impact of different CVD parameters. Recently, Popov et al. [58] proposed an analytical kinetic model of the graphene nucleation and growth in CVD on Cu(111) based on a combination of kinetic nucleation theory and density functional theory (DFT) calculations.

When combined with first principle calculations, the KMC method emerges as a promising approach that is capable of characterizing graphene growth with atomistic resolution and for significantly larger time and length scales compared to other models. Such multiscale models were developed by, e.g., Li et al. [53], who used the Bortz–Kalos–Lebowitz (BKL) algorithm for identifying the dominant pathways of graphene growth in CVD on Cu(111) by focusing on the attachment/detachment of carbon-containing species. The authors applied a mean field approximation for the (de)hydrogenation reactions on the surface to accelerate the KMC simulations by recording the number of hydrogen adatoms and modifying the hydrogenation reaction rates, correspondingly. The study succeeded in identifying the dominant feed species (i.e., C, C${}_{2}$) in growth pathways under different H${}_{2}$ pressures. In another study, Chen et al. [55] proposed an all-atom KMC model considering a simplified reaction list, including reactions of the carbon monomer and its dimer on the surface and the edges, such as ring closure reactions, that are essential for the hexagon formation on the edges. They considered a deposition flux for carbon species instead of explicitly considering the decomposition of CH${}_{4}$ and H${}_{2}$ as precursors in the CVD. Their KMC model predicted different morphologies of growing graphene depending on the deposition flux and temperature profiles. This study confirms the result reported by Wu et al. [59], who showed that the dominant feeding species for graphene growth is C${}_{2}$. Both previous studies were capable of exploring the growth and etching of graphene with a maximum size of simulated flakes of less than 10 nm [55,60,61,62]. Kong et al. [63] developed a large-scale KMC method to investigate graphene growth up to the size of micrometers. To make this possible, they represented carbon attachment and detachment processes by adding or removing entire hexagons to form the edge of a graphene island. The authors reported a complementary relationship of growth and etching of a graphene island, and they also reported the formation of holes in the graphene flake.

Despite all these outstanding efforts to model the growth of graphene for isolated flakes, important questions, concerning the impact of the kinetic pathways on the atomistic scale of reactions involved, remain open. Developing KMC methods that are capable of providing insight into the atomistic scale requires consideration of a complete list of reactions, which was previously limited to the size of the simulated graphene flakes [55,60,61]. For the problem at hand, a full description is complicated severely by the separation of time scales of the various processes. The most dramatic separation of time scales arises between fast diffusion versus slow chemical reactions. While there is a plethora of prior studies, which used reaction rates computed by DFT to model some aspects of graphene growth, they are either limited to very small systems, forced to simplify the model to eliminate the fast process, or they simplify the reactions. One common approximation is to leave out the diffusion of the molecular precursors on the surface completely or, as already pointed out, to combine all separate reactions, in the growth of a new graphene hexagon, into a single reaction [63]. As we will show below, these approximates have significant quantitative and qualitative consequences to model graphene growth.

In this paper, we report a DFT-based KMC model capable of dealing with a comprehensive list of reactions, ranging from the dissociative adsorption of CH${}_{4}$ and H${}_{2}$ as precursors, to edge attachment and detachment of all movable species, as well as ring closure at the edges and the hydrogenation and dehydrogenation of species attached to the edges. A fast implementation of the inherently sequential KMC procedure enables us to include all reactions at the atomistic level while still reaching mesoscopic length scales. Specific consideration of (de)hydrogenation allows for the investigation of the graphene quality as a result of changes in the number of hydrogen-saturated edges. The use of several ratios of precursor partial pressures and numerous relevant reactions (55 reactions) permits the study of the growth pathways and the role of CVD control parameters during graphene growth on a Cu(111) surface. The model developed provides useful insights into the growth mechanism during the steady-state in terms of distinguishing the role of particular reactions, observing the effects of selected CVD control parameters, and estimating the growth rate.

## 2. Results and Discussion

In order to analyze the growth mechanism of graphene, we utilized the density functional theory (DFT) method to determine the activation energy barriers for relevant possible reactions involving the species of interest, i.e., H, C, CH, CH${}_{2}$, CH${}_{3}$, CH${}_{4}$, C${}_{2}$, C${}_{2}$H, and C${}_{2}$H${}_{2}$. Then, we used the list of barriers as an input for the KMC protocol to study the effects of CH${}_{4}$ partial pressure and the role of each reaction in the graphene growth process. Furthermore, we calculated (de)hydrogenation reactions, occurring at the edges during graphene growth, and investigated their impact on the quality of the growing graphene flake using KMC simulations.

### 2.1. Activation Energy Barriers

**Figure 1:**
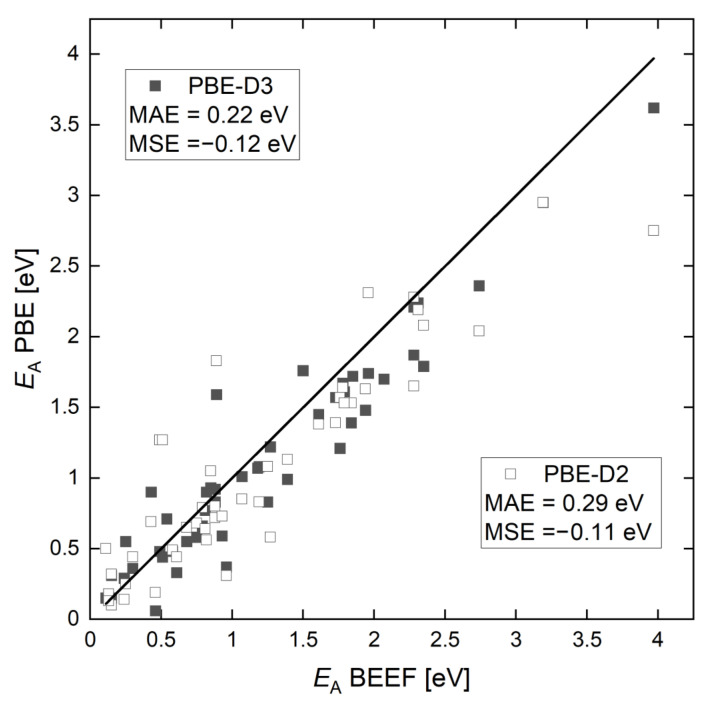
Parity plot for the reaction barriers computed with BEEF-vdW vs. PBE-D3 and PBE-D2 [53].

Since Li et al. reported activation energy barriers of the set of reactions happening during the CVD of graphene on Cu(111) using CH${}_{4}$ and H${}_{2}$ as precursors [64], we firstly analyzed the impact of the DFT method choices on the energy values. As pointed out in our previous study, we found strong co-adsorption effects on the activation energy barrier, especially for reactions on the slab edge [58]. In particular, the activation energy barrier of C${}_{2}$ attachment to the edge (one of the three most crucial reactions in the growing process) was doubled with an increase of the supercell, i.e., 1.22 eV instead of 0.58 eV, which was reported by Li et al. [53]. Therefore, we recalculated all the energy barriers (reaction barriers are listed in Table S1). We compared the activation energy barriers obtained with the BEEF-vdW and PBE-D3 functionals with regard to the literature data and show the results in Figure 1.

The BEEF-vdW functional makes it possible to determine the barrier height for reactions on surfaces very accurately, since it was especially designed for the processes on surfaces [65]. At the same time, other GGA functionals, such as the PBE, tend to underestimate the reaction barriers. We can also observe a similar trend for the reactions considered in this work. For most reactions, the energy barriers, obtained with the BEEF-vdW functional, were higher than for the PBE functional (Figure 1). This is, in particular, noticeable for the detachment reactions, such as the dehydrogenation reactions of methane (Table S1). Therefore, higher barriers with the BEEF-vdW functional, i.e., up to approximately 0.5 eV, were observed.

We found a reasonable agreement between the results obtained by the PBE-D3 and PBE-D2 (Figure S3), but a mean absolute error (MAE) of 0.21 eV still appeared. This was close to the typical error of DFT, but it was larger than expected by just changing the treatment of the dispersion interactions. We assume that the major reason for this discrepancy originated from the different setups for reactions at the graphene edges, i.e., our results included corrections for the co-adsorption error that were not present in the previously reported data (PBE-D2) [53]. This led to a huge difference in the C${}_{2}$ attachment barrier, as mentioned above. We have to note that reaction barriers, where C is involved, were substantially lower in our work than in comparison to the work of Li et al. [53]. This was due to the fact that we did not include any subsurface carbon (since the carbon solubility on copper is very low [66]). Therefore, we found that the attachment barrier of C to the edge was much lower with the PBE-D3 (0.44 eV) than the one with the PBE-D2 (1.27 eV) reported by Li et al.

Using the activation energy barriers and reaction energies obtained, we calculated the linear scaling relations for the detachment of “small species” from the graphene edges (Figure S4). We also calculated linear scaling relations for the dehydrogenation reactions at the graphene edges (Figure S5). For that, we used the so-called transition state scaling relations [67], and the relations we derived are described in the SI. In general, linear scaling relations are an important concept in heterogeneous catalysis [68,69,70,71] that make it possible to predict the activation energy barrier without explicitly calculating the transition state. We see linear scaling relations for the reactions considered, i.e., one can predict the energy barrier for, e.g., the attachment/detachment of CH${}_{3}$ to graphene, by the calculation of the reaction energy of CH${}_{3}$ attachment on the edge without ever calculating the transition state for such a reaction.

Finally, we have to mention that we also calculated the hydrogenation and dehydrogenation reactions, which were missing in the previously reported approach. We have added these reactions to the list of reactions used in the KMC method (see Table S2). They are important because they control hydrogen termination on the flakes, which prohibits graphene growth in the case that these reactions are absent. The impact of (de)hydrogentation reactions on the graphene growth is discussed further.

### 2.2. Modulation of Graphene Growth by CH${}_{4}$ Partial Pressure

In order to study the mechanism of graphene growth on a Cu(111) surface, we utilized the KMC protocol with reaction rates obtained from the DFT as parameters. Eight different simulations were carried out to examine the impact of partial pressures on the rates of chemical reactions occurring during the growth process (see Table 1).

Each simulation resulted in a non-equilibrium steady-state being reached at approximately $2\times 10^{-3}$ s. The concentration of reactive species at this steady-state fluctuated around their respective moving averages, which were observed through concentration plots (Figure S9). In the concentration plots, we found that the most abundant carbon-containing species was the carbon dimer, C${}_{2}$, which had a relaxation time of 2 × 10${}^{-3}$ s (see brown curves in Figures S7 and S8). In addition, we observed that the concentration of hydrogen was usually higher than the concentration of other carbon-containing species. This was due to the dissociative adsorption rate of H${}_{2}$, which is higher than that of CH${}_{4}$. This happened even when the CH${}_{4}$ partial pressure was several orders of magnitude higher than the H${}_{2}$ partial pressure. Moreover, we found that increasing the CH${}_{4}$ partial pressure (for a fixed H${}_{2}$ partial pressure) resulted in an increase in carbon and carbon dimer concentrations (see Figure S9a). Conversely, increasing the H${}_{2}$ partial pressure (for a fixed CH${}_{4}$ partial pressure) led to an increase in hydrogen adatom concentration and a decrease in carbon and carbon dimer concentrations (see Figure S9b).

**Figure 2:**
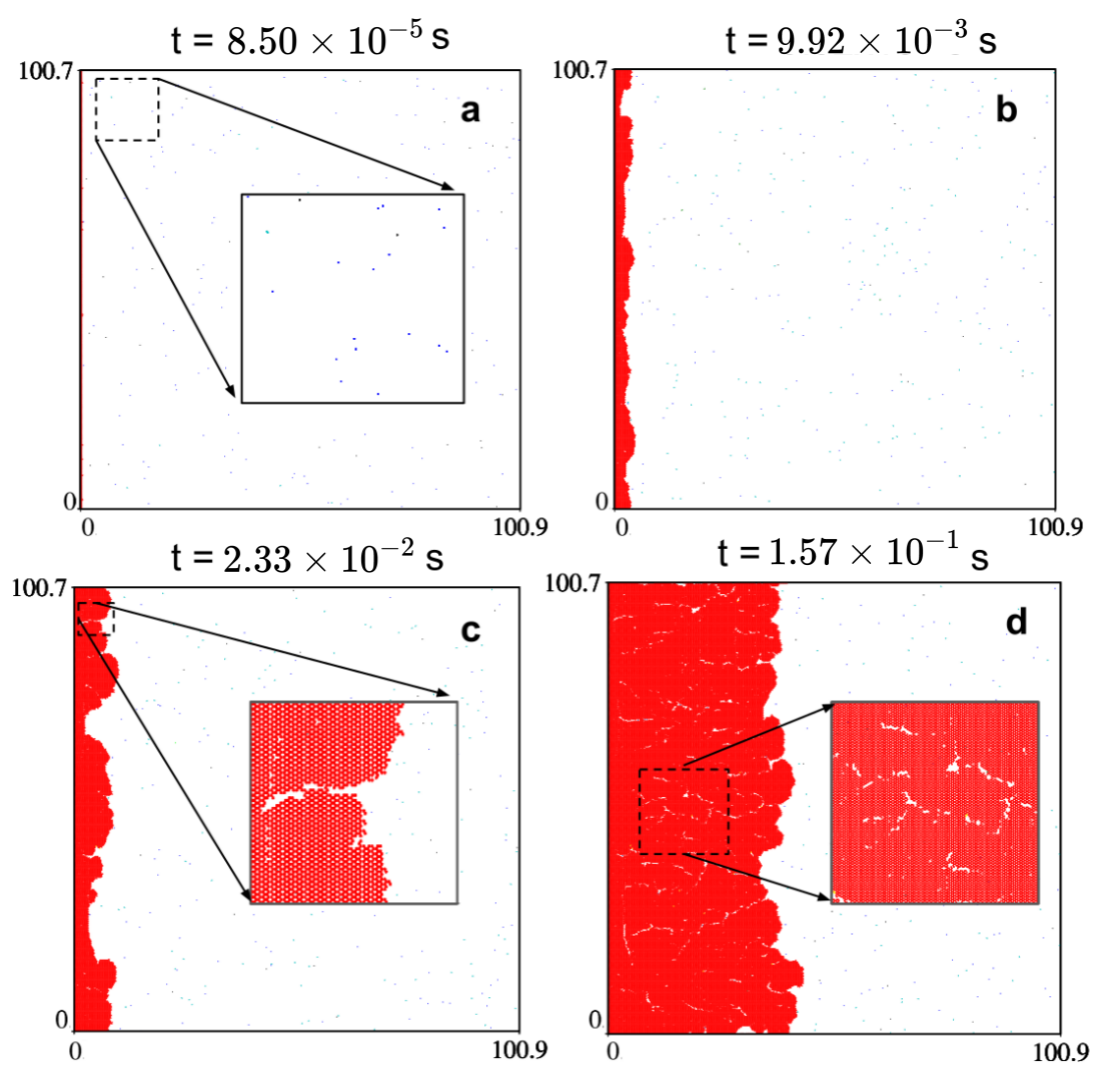
KMC simulation of CVD graphene growth on Cu(111). The Cu(111) surface is represented by 100 × 100 nm${}^{2}$ lattice for P${}_{CH_{4}}$ = 100 Torr, P${}_{H_{2}}$ = 0.01 Torr, and T = 1300 K (sample S1). Each snapshot has a timestamp and shows a different stage of the growth process. (**a**) Initial stage of growth, where hydrogen- and carbon-containing species adsorb on the surface due to the CH${}_{4}$ and H${}_{2}$ dissociative adsorption and decomposition. (**b**) The flake growth after reaching a steady state, i.e., at around 2 × 10${}^{-3}$ s. (**c**,**d**) Growth stages at 2.33 × 10${}^{-2}$ s and 1.57 × 10${}^{-1}$ s, respectively. The formation of cracks and irregular edges, caused by vacancy defects and hydrogenation at the edges, are visible in the inset.

Figure 2 demonstrates qualitatively the spatio-temporal evolution of the sample S1 (Table 1), i.e., we show snapshots from the KMC simulations. Similar evolution snapshots for other samples are given in the SI. Due to the CH${}_{4}$ and H${}_{2}$ adsorption and their subsequent decomposition on the surface, different species were formed with increasing concentrations over time (Figure S7a). After approximately 2 × $10^{-3}$ s, we observed that the system reached its steady-state (see Figure S7a). This permitted the start of the flake growth via the attachment of species to the edge (Figure 2b). Since the width of the initial and growing flake was around 100 nm, different species could attach at different positions simultaneously, which also resulted in vacancy defects (see the inset plots in Figure 2c,d).

**Figure 3:**
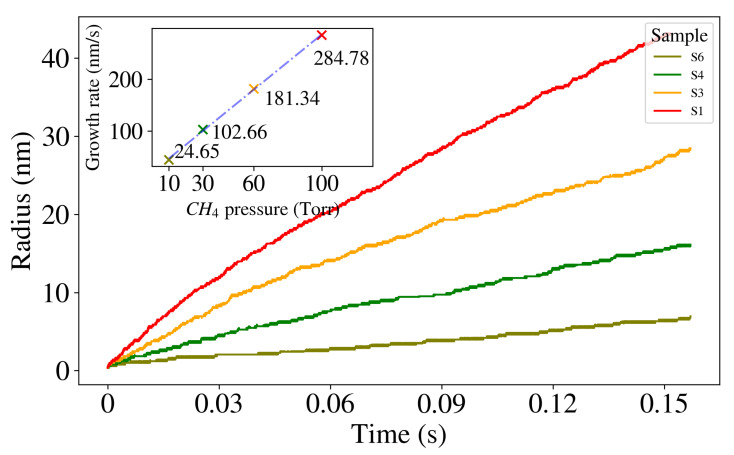
Time series of graphene growth for different CH${}_{4}$ partial pressures. The change in the flake radius for samples S1 (100 Torr), S3 (60 Torr), S4 (30 Torr), and S6 (10 Torr) are shown with the red, orange, green, and olive color, respectively. The KMC simulations up to 0.157 s were performed, and the resulting flake radii of approximately 38 nm, 24 nm, 12 nm, and 4 nm, respectively, were obtained. Inset plot: Flake growth rate (at 0.157 s of KMC simulation) as a function of the CH${}_{4}$ partial pressure profile. The blue dashed line shows the fitted curve on data, thereby indicating a linear relation between the flake growth rate and CH${}_{4}$ partial pressure.

We conducted a comprehensive analysis of the spatio-temporal evolution of all the samples (refer to Figures S10–S16). Our findings suggest that graphene growth occurs at a faster rate with a higher partial pressure of CH${}_{4}$, while it slows down with an increase in H${}_{2}$ partial pressure. These observations are consistent with previously reported studies, which also observed the impact of CH${}_{4}$ partial pressure on flake size and graphene quality [29,39,42,72]. To gain further insight into the growth mechanism, we plotted the time dependence of the radius of the growing flake during the simulation (refer to Figure 3). Here, we defined the flake radius (mean radius) as the average of the horizontal distance between the flake’s outer layer edges and the vertical axis (schematically depicted in Figure S23). Summary on the mean radius (R${}_{mean}$) and simulated time for all samples is shown in the Table S3. The results clearly indicate that increasing the partial pressure of CH${}_{4}$ (while keeping the H${}_{2}$ partial pressure constant) leads to the growth of larger graphene flakes. This happens because a higher concentration of carbon-containing species results in an increased number of successful attachments. The curve fitting yielded a linear dependency between the growth rate, which was calculated at the KMC simulation of 0.157 s, and the CH${}_{4}$ partial pressure as (see inset plot in Figure 3):

(1) $$R=2.66\times P_{CH_{4}}+20.38.$$

It should be noted that the instantaneous growth rate decreases over time (see Figure S21 in SI). It is connected to the fact that the number of available adsorption sites decreases with time. To evaluate the quality of the graphene flakes grown on the Cu(111) surface, we calculated the surface average roughness (R${}_{a}$) and the root-mean-square roughness (R${}_{q}$) using Equations (S6) and (S7) in the SI. We observed that increasing the CH${}_{4}$ partial pressure during the growth process resulted in different radii of the flakes due to a varying concentration of carbon-containing species and the number of successful attachments. This variation in the radii of the flakes led to an increase in the surface roughness, as demonstrated in Table 2. Therefore, we conclude that the surface roughness of the flakes is influenced by the partial pressure of CH${}_{4}$ during the growth process.

Finally, we observed a high concentration of H adatoms (i.e., 4 ×$10^{-4}$, see Figure S9), particularly for samples with low CH${}_{4}$ partial pressure, such as S2, S5, S7, and S8. This finding is consistent with the results of experiments in [73], where it was suggested that the dissociative adsorption of CH${}_{4}$ on Cu(111) was less favorable compared to the dissociative adsorption of H${}_{2}$, thus resulting in a higher concentration of H adatoms relative to the carbon-containing species.

### 2.3. The Growth Mechanism

With this study, we also wanted to identify the relevance of the individual reactions involved in graphene growth and determine the specific pathways leading to its formation. To achieve this, we calculated 55 reactions using DFT (see Table S2) and used them in the KMC method. Some of reactions were not studied in previous works, e.g., [53,74] (for comparison, see Table S4). We recorded the occurrence frequency of each reaction during the simulation of different samples and we observed, in general, similar trends (see Figures S18 and S19) variations due to the CH${}_{4}$ and H${}_{2}$ partial pressures.

**Figure 4:**
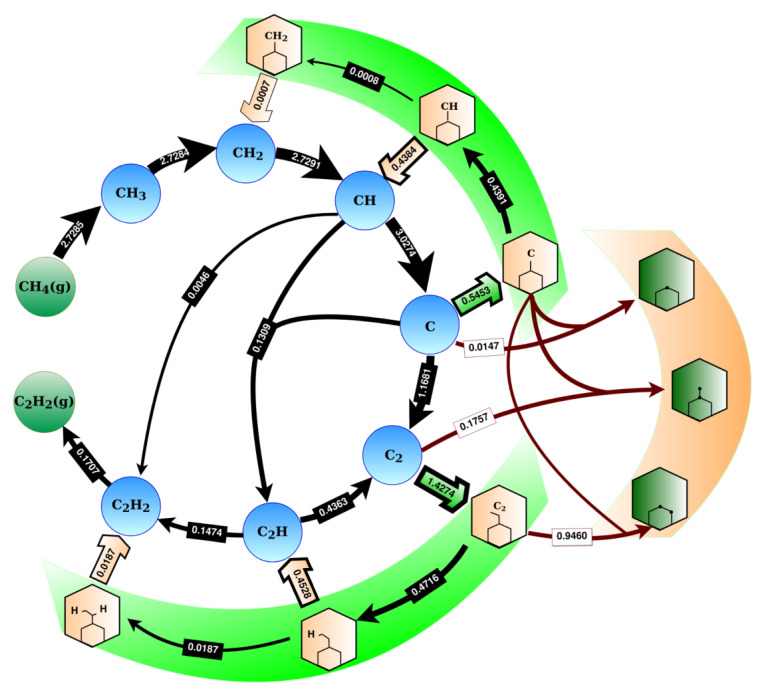
Occurrence map of elementary reactions involved in the graphene growth process on Cu(111) at P${}_{CH_{4}}$ = 100 Torr, P${}_{H_{2}}$ = 0.01 Torr, and T=1300 K (sample S1, see Table 1). The map summarizes net contributions (per second per site) of the most relevant events in the KMC simulation for 0.138 s. Blue and green circles indicate free species on the lattice. Green highlighted regions represent attachment of species to the flake edges, while brown highlighted region shows the hexagon formation via ring closure reactions on the edges. H${}_{2}$ dissociative adsorption and desorption, as well as diffusion of species, are not shown here. Gas phase species are marked with a “*g*”. The possible conversions are shown as the arrows in the direction of the net contribution (forward minus backward occurrences). The numbers on arrows are the net contribution per second per site.

In this section, we focus on reaction pathways and their contributions to the growth process occurring in sample S1 (with P${}_{CH_{4}}$ = 100 Torr, P${}_{H_{2}}$ = 0.01 Torr, and T = 1300 K), which served as a representative example. The corresponding occurrence map is presented in Figure 4 (and Figure S17 in SI), where we identified three main regions representing different groups of reactions: those involving free species (excluding hydrogen adatom reactions), edge attachment reactions, and growth reactions, which are denoted by blue (and green) circles, highlighted in green and brown, respectively.

Following the sequence in the occurrence map, we can analyze the net contribution direction (forward arrows) in detail. The precursor used in this study is CH${}_{4}$, which decomposes into CH${}_{3}$ and CH${}_{2}$ on the Cu surface, to result in the formation of CH (2.7291 events/s/site). From CH, there are three main reaction pathways: (1) decomposition into single carbon (3.0274 events/s/site), (2) C${}_{2}$H formation (0.1309 events/s/site) and (3) C${}_{2}$H${}_{2}$ formation (0.0046 events/s/site). In general, the occurrence of events in these pathways directly depends on the activation energy barriers of the particular reaction (see Table S2) and the concentration of species (see Figure S9a). Therefore, the reactions with the lowest activation energy and the highest concentration of species occur most frequently. From Figure 4, we see that the net contribution of these reactions results in the formation of a carbon monomer, which is the building block for graphene growth. The most frequent reaction in the free species region that directly contributes to graphene growth is C${}_{2}$ formation (1.1681 events/s/site), which involves two single carbon atoms. Moreover, the carbon monomer also participates in C${}_{2}$H formation (0.1309 events/s/site) and in the growth of graphene via attachment to the edge (0.5453 events/s/site) or the hexagon formation (0.0147 events/s/site), see Figure 4. Therefore, the formation of a single carbon occurs more frequently than the subsequent C${}_{2}$ formation (by ca. 721,797 times), which has a significant impact on the concentration of both species during the growth process (see Figure S9).

Moving on to the next set of reactions, C${}_{2}$ plays a crucial role in the growth of graphene. It participates in several reactions, such as carbon dimer attachment to the edge (1.4274 events/s/site), ring closure reactions (0.1757 events/s/site), and hydrogenation reaction to form C${}_{2}$H (0.4716 events/s/site). Notably, our study revealed that C${}_{2}$ contributes more towards graphene growth than the carbon monomer. This highlights the importance of both species in graphene formation. However, the formed C${}_{2}$H species can decompose back into C${}_{2}$ (0.4363 events/s/site), thus leading to an increase in the content of C${}_{2}$. C${}_{2}$H${}_{2}$ is the last species in the free species region and can either desorb from the surface (0.1707 events/s/site) or decompose into CH. Additionally, dehydrogenation reactions from the edges (0.0187 events/s/site) increase the final contribution in C${}_{2}$H${}_{2}$ desorption. The net contributions of reactions in the free species region are influenced by reactions on the flake edge, which include the (de)hydrogenation of attached species, as well as the attachment and detachment of species to the edge (reactions 19–29 in Table S2). These edge attachment reactions are highlighted in green in Figure 4.

The contribution of edge attachments of carbon and its dimer were found to be the most stable, while other attached species were more likely to be hydrogenated and detached, as indicated by the arrows in Figure 4. We discovered that the hydrogenation of attached species at the edges was more favorable than their dehydrogenation. This led to a positive net contribution towards hydrogenation for all attached species, particularly in the green highlighted region in Figure 4. As a result, there was a competition between hydrogenation at the edges and detachment reactions from the edges, which had not been previously studied in the literature. The net contribution of H${}_{2}$ in the desorption direction was determined to be approximately 5.1519 events/s/site. This indicates that some hydrogen adatoms leave the surface, while the remaining species participate in other reactions, such as hydrogenation. These (de)hydrogenation reactions must be included in the reaction set used in the KMC simulation, as they significantly impact the quality of the graphene flake. Further details on all reactions involved in the process are provided in the Supplementary Information (Figure S20). The analysis shows that there were similarities in the reaction pathways towards graphene growth, as we described for sample S1. At the same time, net contributions for forming C-containing species, attachment to the edges, and ring closure reactions were higher for samples with higher methane partial pressures (S1, S3, and S4 from Table 1) due to the increased CH${}_{4}$ partial pressure. This will be discussed in more detail in the next section.

To successfully grow graphene, it is crucial to allow ring closure reactions, which are highlighted by the brown region in Figure 4. The main contribution to growth comes from the rotation of C${}_{2}$ attached to the edge (0.9460 events/s/site). A hexagon ring can be completed either by attaching a C${}_{2}$ to two dangling C on the edge or by attaching three single carbon atoms, with the first two carbon monomers being attached next to each other and the third one completing the ring. The reaction barriers for both processes are listed in Table S2 (number 30–32). By analyzing the occurrence map (Figure 4) and the frequency of events, we can conclude that C and C${}_{2}$ species played the primary role in graphene growth under the considered conditions. This observation is in line with the findings of a recent study that used analytical kinetic modeling to investigate graphene nucleation and growth [58]. The inclusion of ring closure reactions in the reaction set is essential for the accurate simulation of graphene growth pathways, which we will discuss in more detail in the following section. For a comprehensive analysis of all reactions, including the net contributions for different samples, refer to the Supplementary Information (Figure S20). Samples with higher methane partial pressures (S1, S3, and S4 from Table 1) exhibited higher net contributions to forming C-containing species, attachment to edges, and ring closure reactions due to the higher CH${}_{4}$ partial pressure.

### 2.4. Hydrogenation Reactions

To evaluate the impact of (de)hydrogenation on graphene growth, we conducted KMC simulations with and without the set of (de)hydrogenation reactions for samples S1 (P${}_{CH_{4}}$ = 100 Torr, P${}_{H_{2}}$ = 0.01 Torr) and S2 (P${}_{CH_{4}}$ = 10 Torr, P${}_{H_{2}}$ = 0.001 Torr) at T=1300 K. Due to the lower partial pressure of CH${}_{4}$ and H${}_{2}$ in S2, more time was required to achieve flakes of comparable sizes. As a result, we collected data over 0.161 s and 0.936 s of simulation time to analyze sample S1 and S2, respectively. We conducted a systematic comparison considering three parameters: (1) the ratio of hydrogenated sites over the edges in the flake, (2) the ratio of defects (the number of vacancies) in the actual flake over a defect-free flake with the same average radius, and (3) the surface roughness and root-mean-square roughness parameters of the graphene flake. Table 3 presents a comparison between the ratio of hydrogenated edges and vacancies for samples S1 and S2 with and without (de)hydrogenation reactions. The results demonstrate that incorporating hydrogenation reactions in the simulation of sample S1 led to a decrease of approximately 0.96% in the number of hydrogenated edges, which can hinder proper graphene growth. Additionally, there was a reduction of 2.1% in the number of defects (vacancies) observed in the actual flake compared to the defect-free flake with the same average radius. The difference was even more significant for sample S2, with a reduction of 1.25% in hydrogenated edges and 3.4% in the number of defects. These findings suggest that including hydrogenation reactions in the simulation results for higher-quality graphene growth is important.

**Figure 5:**
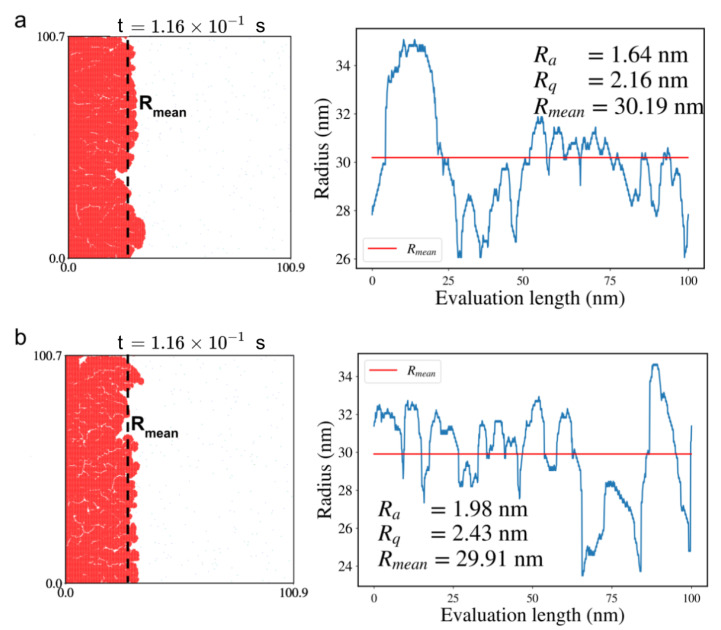
Surface roughness analysis for graphene flake in sample S1: (**a**) with (de)hydrogenation reactions, (**b**) without (de)hydrogenation reactions in the KMC reaction list. On the left: snapshots of the flake at 0.116 s of the simulation (in red) with marked mean radius (R${}_{mean}$) are shown. On the right: the surface roughness plots, as the deviation of radius from mean radius over evaluation length, are given. The red line shows the mean radius (R${}_{mean}$), while the blue curve shows the radius over evaluation length. The average roughness, R${}_{a}$, the root-mean-square roughness, R${}_{q}$, and R${}_{mean}$ are given in the inset for clarity.

**Figure 6:**
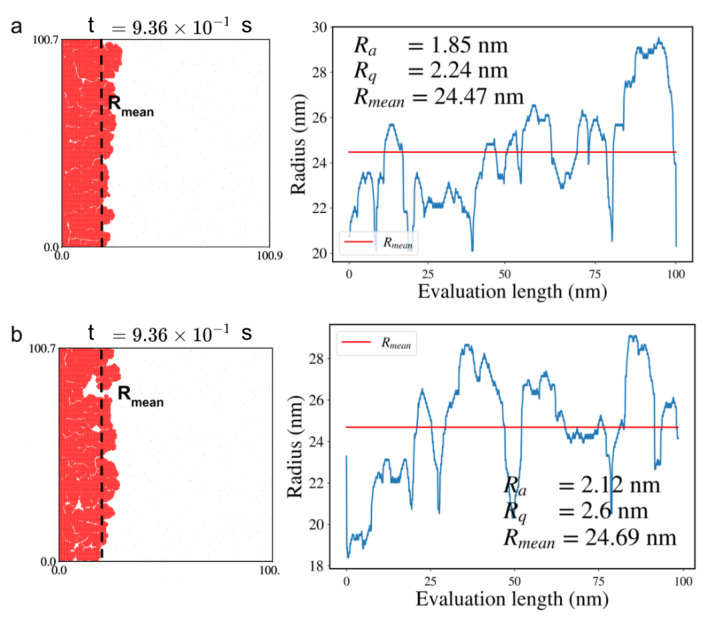
Surface roughness analysis for graphene flake in sample S2: (**a**) with (de)hydrogenation reactions, (**b**) without (de)hydrogenation reactions in the KMC reaction list. On the left: snapshots of the flake at 0.936 s of the simulation (in red) with marked mean radius (R${}_{mean}$) are shown. On the right: the surface roughness plots, as the deviation of radius from mean radius over evaluation length, are given. The red line shows the mean radius (R${}_{mean}$), while the blue curve shows the radius over evaluation length. The average roughness, R${}_{a}$, the root-mean-square roughness, R${}_{q}$, and R${}_{mean}$ are given in the inset for clarity.

We analyzed the surface roughness characteristics of the grown graphene flakes by calculating the mean flake radius (R${}_{mean}$), surface roughness (R${}_{a}$), and root-mean-square roughness (R${}_{q}$) using Equations (S6) and (S7) in the SI. Snapshots of the flakes and their roughness profiles, as well as changes in their radii over the evaluation length, are shown in Figure 5 and Figure 6 for sample S1 and S2, respectively.

The influence of (de)hydrogenation on the quality of the grown graphene is evident from the results. The surface roughness of the samples was substantially lower in the presence of (de)hydrogenation reactions, with values of 1.64 nm and 1.85 nm for S1 and S2, respectively, compared to 1.98 nm and 2.12 nm, respectively, when the reactions were absent. Additionally, the inclusion of (de)hydrogenation reactions led to a decrease in root-mean-square roughness, whose values were reduced by 0.27 nm and 0.36 nm for S1 and S2, respectively. To further analyze the roughness of the samples, we used the radius as a measure and calculated the roughness profile, which shows the deviation of the radius from the mean radius (refer to the snapshots in Figure 5 and Figure 6).

The quality of graphene growth was strongly influenced by the (de)hydrogenation reactions, as demonstrated by the surface roughness analysis and the observed reduction in the number of hydrogenated edges and defects (vacancies). The results suggest that incorporating (de)hydrogenation reactions on the edges leads to higher quality graphene material.

## 3. Materials and Methods

**Figure 7:**
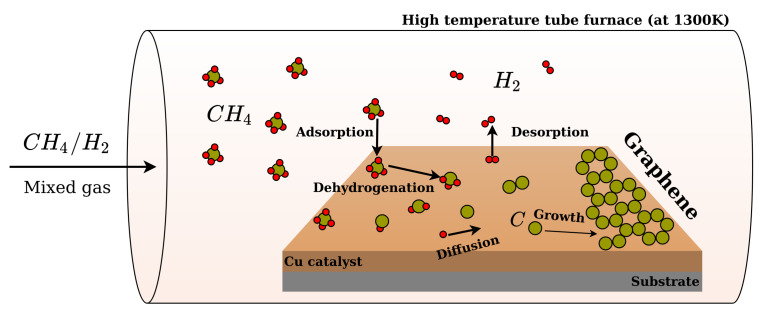
Example of the chemical vapor deposition procedure used for graphene synthesis on Cu as the metal catalyst. The mixture of precursor gases enters the chamber under defined conditions of temperature and gas partial pressures. Various types of reactions, such as adsorption/desorption, (de)hydrogenation, and surface diffusion, happen.

For the development of the multiscale model, we considered the CVD conditions depicted in Figure 7. The chamber of the CVD process contained a mixture of CH${}_{4}$ and H${}_{2}$ gases with constant partial pressures P${}_{CH_{4}}$ and P${}_{H_{2}}$. Generally, in experimental conditions, the gas mixture also contains a carrier gas, e.g., argon, which does not participate in any chemical reaction [72,75]; therefore, it was not taken into consideration here. The tube furnace comprises substrate and catalyst, e.g., a copper plate interfacing with the gas at temperatures in a range of 1000–1300 K. Among possible Cu surfaces, we considered the ideal Cu(111) surface and a temperature of 1300 K. It is important to note that such temperatures are rather close to the melting point of copper (1357 K), which means that surface Cu atoms can be quite mobile. Although there are some theoretical studies in the literature indicating the impact of the mobility of Cu atoms on the coalescence process [76], we neglected these effects in the present work and considered a solid, rigid surface. We used the adsorption rate of both gases, which depends on their partial pressures, system temperature, and dissociative adsorption activation energies (more details in the SI).

### 3.1. DFT Calculations and Reaction Rates

All quantum mechanical calculations were performed using density functional theory with the BEEF-vdW [77] and PBE [78,79,80,81]-D3 [82] functionals as implemented in the Vienna ab initio simulation package (VASP 5.4) [83,84]. The projector augmented wave method (PAW) method [85] for the description of the core electrons was employed. A kinetic energy cutoff for the plane wave expansion was set to 450 eV. The energy convergence threshold of $10^{-6}$ eV was used. The optimized Cu bulk lattice constants of 3.56 Å and 3.64 Å were obtained using the PBE-D3 and BEEF-vdW functionals, respectively.

To calculate reactions between “small species”, e.g., the dehydrogenation of methane, the Cu(111) surface was modeled by a four-layer 3 × 3 slab (with 2 bottom layers being frozen), see Figure S1. The surface was modeled as a three-layer slab (10 × 3) with two bottom layers being frozen (see Figure S1) for calculations of graphene edges. For the latter, a 5-ring-wide graphene ribbon, depicted in Figure S2, was used. The relaxation of the upper layers and adsorbed hydrocarbon species was employed with the conjugate gradient method until the total energy change between two ionic relaxations was smaller than $10^{-5}$ eV. A vacuum region of at least 12 Å perpendicular to the surface was used. The Brillouin zone was sampled by a 6 × 6 × 1 for the 3 × 3 slab and a 2 × 4 × 1 Monkhorst–Pack k-point mesh for the calculations involving graphene edges to provide sufficient accuracy. All barriers of attachment were calculated with respect to individualized species, as explained in Ref. [58].

In the present study, we have not considered subsurface processes. However, we included five reaction barriers for the formation and reconstruction of the hexagon at the graphene edge, which were proposed by Chen et al. [55]. Transition states were determined with the nudge elastic band (NEB) method [86]. All calculations were conducted according to the non-spin-polarized scheme.

### 3.2. KMC Model

**Figure 8:**
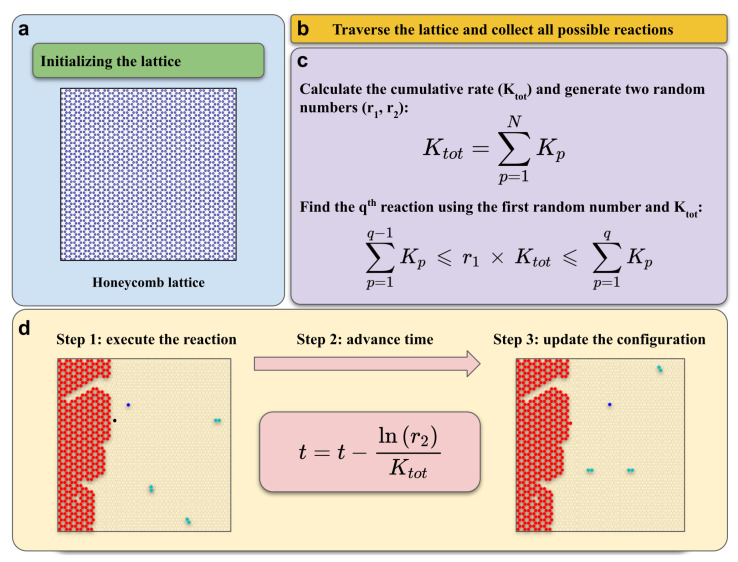
The KMC protocol implemented in the multiscale model. (**a**) The honeycomb lattice used in the model. The collection (**b**), selection (**c**), and update (**d**) schemes. An example of the change of the state configuration during graphene growth is depicted in updating scheme. The description of the algorithm is given in the text.

To efficiently simulate CVD growth processes with elementary steps characterized by diverse rates, we developed an algorithm based on the rejection-free KMC approach, known as the BKL algorithm [87,88]. We applied the KMC protocol to the Cu(111) surface and mapped it to a honeycomb lattice constructed from fcc and hcp adsorption sites. Lattice vector and the nearest neighboring distance were set to 0.246 nm and 0.142 nm, respectively (see Figure S6). The KMC protocol has three main parts (see Figure 8): (i) a **Collection scheme**, in which the algorithm traverses the lattice points to collect species and, after considering their neighboring sites and the reference reactions list, collects all possible reactions from current configuration as a list; (ii) a **Selection scheme**, where the algorithm calculates the cumulative (total) rate of all possible reactions collected in the previous step and generates two random numbers (the algorithm multiplies the first random number by the cumulative rate to select a reaction from the possible reaction list); and (iii) an **Update scheme** with three stages, which include executing the selected reaction based on its type, advancing the simulation time using the second random number generated before, and updating the lattice configuration accordingly.

We calculated all reaction rates, except for the CH${}_{4}$ and H${}_{2}$ adsorption, according to the transition state theory (TST) [89] using activation energy barriers obtained by DFT calculations (see Section 3.1). To calculate the adsorption rates for CH${}_{4}$ and H${}_{2}$, we used the ideal gas approximation [53] considering dissociative adsorption barriers, pressures, the number of available sites on the lattice, and system temperature (1300 K) (see Section S3 in SI). Table 4 contains adsorption rates, where P${}_{CH_{4}}$, P${}_{H_{2}}$, and $N_{free}$ denote the pressure of CH${}_{4}$, the pressure of H${}_{2}$, and the number of free sites on the lattice, respectively. These rates include not only the effect of pair pressures on adsorption of the gases, but also on-the-fly modification of rates due to the change in the number of available sites during simulations.

One of the main aims of this study was to determine how the growth of graphene using CVD is affected by the partial pressures of CH${}_{4}$ and H${}_{2}$. To accomplish this objective, we generated a list of precursor partial pressure pairs and created corresponding samples for our investigation. The samples were then categorized into two primary profiles based on the partial pressures of CH${}_{4}$ and H${}_{2}$ at T = 1300 K. We set the H${}_{2}$ pressure to 0.01 Torr and varied the CH${}_{4}$ pressure to 10, 30, 60, and 100 Torr for the CH${}_{4}$ partial pressure profile. For the H${}_{2}$ partial pressure profile, we fixed the CH${}_{4}$ pressure at 10 Torr and varied the H${}_{2}$ pressure to $10^{-3}$, 5 ×$10^{-3}$, 5 ×$10^{-2}$, and 8 ×$10^{-2}$ Torr. We labeled these pairs of pressure sets as samples shown in Table 1. Note that CH${}_{4}$ and H${}_{2}$ partial pressures used in this study may not be exactly comparable with experiments. Relatively high theoretical CH${}_{4}$ pressure would be required to practically take into account the imperfection of the experimentally employed Cu substrate, where defects provide stronger binding energy, thereby leading to higher adsorption rates of precursors. Therefore, we intentionally chose a range of partial pressures that exceeded experimental values. This approach ensured that adsorptions occurred more frequently, especially in scenarios where other rate constants were much higher than adsorption. With this in mind, we carried out KMC simulations for different pressure profiles to study various pathways of graphene growth. Our simulations began with an initial flake in the form of a ribbon or slab, which was located on the left side of a 100 × 100 nm${}^{2}$ lattice containing 388,206 adsorption sites.

## 4. Conclusions

To study the CVD growth of graphene on a Cu(111) surface at 1300 K, we developed a multiscale model by combining first principle calculations and the KMC method. By implementing a kinetic Monte Carlo program, we were able to accurately represent relevant atomic scale reactions without any additional approximations, thereby allowing us to study long time and length scales. Specifically, we were able to observe graphene flakes of up to 38 nm that formed in 0.157 s under different CH${}_{4}$ and H${}_{2}$ partial pressures. Our investigations of various synthesis conditions revealed that the growth rate (flake radius per time) was primarily dependent on the CH${}_{4}$ partial pressure, which varied linearly from 24.65 nm/s at 10 Torr to 284.78 nm/s at 100 Torr. For systems with a constant CH${}_{4}$ partial pressure, the growth rate remained relatively consistent, ranging from approximately 41 to 48 nm/s across different H${}_{2}$ pressure profiles. We also found that samples with a higher CH${}_{4}$ partial pressure had faster growth rates, thus indicating that the partial pressure ratio of CH${}_{4}$:H${}_{2}$ played a crucial role in the growth mechanism (refer to Figure S22). Specifically, the higher methane partial pressure led to a higher concentration of carbon-containing species on the lattice, which accelerated the growth process.

We also identified the role of individual reactions that occurred during graphene growth and studied the reaction pathways in detail. Data analysis suggested that the carbon monomer (C) and dimer (C${}_{2}$) have the highest contribution to graphene growth. C${}_{2}$ was found to be the dominant feeding species during growth under the conditions considered, which is consistent with previous studies [53,54,55,59]. Overall, our method allowed us to gain a deeper understanding of the growth mechanism and the influence of synthesis conditions on the growth rate. Several validation simulations showed that it is important to consider the reactive precursors in the growth process explicitly. Due to strong co-adsorption effects, the activation barrier impacts the growths mechanisms, i.e., in the case reported here, the attachment of C${}_{2}$ to the graphene zigzag edge was effectively twice what was previously reported [53]. Moreover, the attachment barrier of C was substantially different in our calculations. Because the activation barrier entered the reaction rate exponentially, this may have led to a qualitative change in the growth mechanism and the relative importance of the role of reacting species, as was demonstrated in our study in comparison to prior work [53]. Finally, the implementation of (de)hydrogenation reactions of species on the graphene edge in the multiscale model permitted us to investigate its impact on the quality of the grown graphene for the first time. Here, surface roughness and the content of hydrogenated and defective (vacancy) sites were shown to be dependent on the synthesis conditions and the presence of (de)hydrogenation, as was also reported by Sun et al. [90] from experiments.

The multiscale method discussed in this report offers a valuable perspective on the CVD growth of graphene on Cu(111), thus providing insights into how controlling parameters affect the quality of the material. This method is particularly helpful for understanding the kinetics of CVD graphene growth and other experimental observations at the relevant temperatures and pressures used in the production of graphene. However, it is important to note that our model assumed a hexagonal lattice, which limited the growth mechanism to hexagonal symmetry. This means that we cannot model topological defects such as rings of atoms with five or seven atoms. Additionally, our simulations began with an initial flake as a ribbon (measuring 0.284 × 100 nm${}^{2}$) located on the lefthand side of the lattice, so we did not consider graphene nucleation. Furthermore, we assumed a perfect, fixed Cu(111) substrate and a monolayer of graphene, as most experimental studies aim to produce high-quality monolayer graphene sheets with uniform properties that are comparable to exfoliated graphene [91]. We did not account for defects in the copper substrate or doping by other atoms, such as nitrogen, as has been reported in previous studies [60,92]. Therefore, the method developed here cannot simulate a wide range of possible experimental conditions. The inclusion of defects is an important direction for future research, but it would require the calculation of the energy barriers for a multitude of reactions, which is beyond the scope of the current study. Additionally, it is worth noting that the current version of our KMC code was limited to single-core usage, thereby making it infeasible to simulate larger lattice sizes. However, further parallelization of the code could help overcome this limitation. 

## Figures and Tables

**Table 1:** The list of the pressure setup used for the KMC simulations (values in Torr).

Sample	P${}_{{\mathrm{CH}}_{4}}$	P${}_{H_{2}}$
S1	100	0.01
S2	10	0.001
S3	60	0.01
S4	30	0.01
S5	10	0.005
S6	10	0.01
S7	10	0.05
S8	10	0.08

**Table 2:** The surface roughness parameters, R${}_{a}$ and R${}_{q}$, with the average radius, R${}_{mean}$, for increasing CH${}_{4}$ partial pressure profile.

Sample	R${}_{\mathrm{mean}}$ (nm)	R${}_{a}$ (nm)	R${}_{q}$ (nm)
S6	3.88	1.24	1.51
S4	13.14	1.3	1.63
S3	25.14	1.31	1.69
S1	38.51	1.73	2.22

**Table 3:** The ratio of hydrogenated edges and defect-containing sites with regard to all edge and body sites in the graphene flake (as a percent). The KMC simulations with (the subscript ’w’) and without (the subscript ’w/o’) (de)hydrogenation reactions of samples S1 and S2 are considered.

%	S1${}_{w}$	S1${}_{w/o}$	S2${}_{w}$	S2${}_{w/o}$
Hydrogenated edge	0.44	1.39	0.15	1.38
Vacancy defect	5.7	7.8	6.8	10.2

**Table 4:** Adsorption rates of CH${}_{4}$ and H${}_{2}$ (in $s^{-1}$).

$r_{H_{2}}$	$r_{{\mathrm{CH}}_{4}}$
$2170\times p\left(H_{2}\right)\times N_{free}$	$0.0329\times p\left({\mathrm{CH}}_{4}\right)\times N_{free}$

## Data Availability

The datasets generated during and/or analyzed during the current study are available from the corresponding author upon reasonable request.

## Supplementary Materials

The following supporting information can be downloaded at: https://www.mdpi.com/article/10.3390/ijms24108563/s1. Refs. [93,94,95,96,97,98] are cited in the Supplementary Material.

## Abbreviations

The following abbreviations are used in this manuscript:

2D	Two-Dimensional
CVD	Chemical Vapor Deposition
PECVD	Plasma-Enhanced Chemical Vapor Deposition
KMC	Kinetic Monte Carlo
DFT	Density Functional Theory
VASP	Vienna Ab Initio Simulation Package
BKL	Bortz–Kalos–Lebowitz algorithm
PBE-D3	Perdew–Burke–Ernzerhof Functional with Grimme D3 Dispersion Correction
BEEF-vdW	Bayesian Error Estimation Functional with van der Waals Correlation
PAW	Projector-Augmented Wave Method
NEB	Nudge Elastic Band Method
TST	Transition State Theory
MAE	Mean Absolute Error
