# Supplementary material for: Multiscale Model of CVD Growth of Graphene on Cu(111) Surface
Source: Int J Mol Sci. 2023 May 10;24(10):8563. doi: 10.3390/ijms24108563 (PMC10217890; doi:10.3390/ijms24108563)
Supplement: Supplementary file 1 [file ijms-24-08563-s001.zip › ijms-2345810-SI.pdf]

# Supplementary Materials: Multiscale Model of CVD Growth of Graphene on Cu (111) Surface

**Meysam Esmaeilpour [1,†], Patrick Bügel [1,†], Karin Fink [1], Felix Studt [2,3], Wolfgang Wenzel [1] and Mariana Kozlowska [1,*]**

## S1. DFT calculations and reaction rates

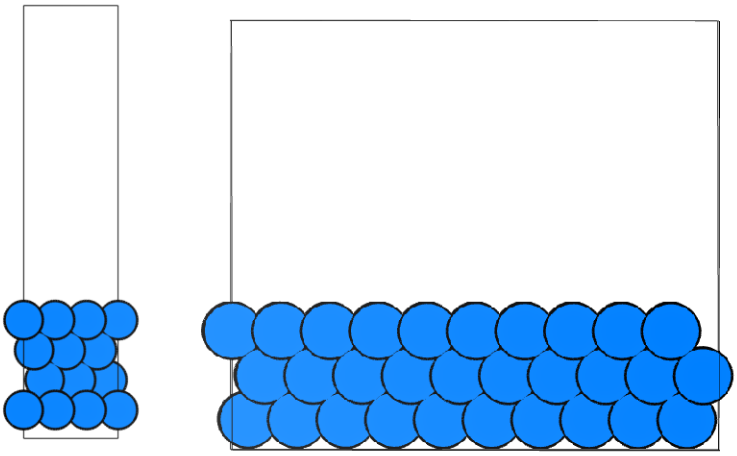

**Figure S1.** Four layer 3 × 3 slab (on the left) and three layer 10 × 3 slab (on the right) that were used to model the Cu(111) surface.

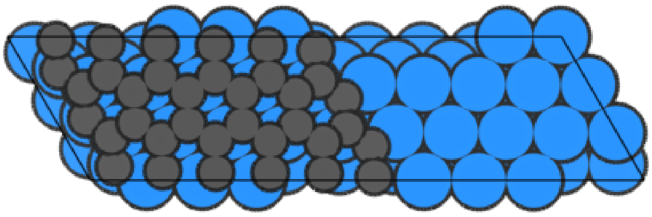

**Figure S2.** 5-rings wide graphene ribbon model that was used for the calculation of all reactions involving graphene.

**Table S1.** The comparison of the calculated activation energies, $E_a$, from the present work (i.e. with the PBE-D3 and the BEEF-vdW functionals) with the activation energies obtained using PBE-D2 functional [1]. All values are in eV.

| Entry | Reaction | Type | $E_a$ (PBE-D3) | $E_a$ (BEEF-vdW) | $E_a$ (Li et al) |
|---|---|---|---|---|---|
| 1 | $H_2 \rightleftharpoons H + H$ | adsorption/desorption | 0.37/0.92 | 0.96/0.88 | 0.31/0.80 |
| 2 | $CH_4 \rightleftharpoons CH_3 + H$ | adsorption/desorption | 1.48/0.90 | 1.94/0.43 | 1.63/0.69 |
| 3 | $CH_3 \rightleftharpoons CH_2 + H$ | detachment/attachment | 1.39/0.58 | 1.84/0.75 | 1.39/0.68 |
| 4 | $CH_2 \rightleftharpoons CH + H$ | detachment/attachment | 0.99/0.55 | 1.39/0.69 | 1.13/0.65 |
| 5 | $CH \rightleftharpoons C + H$ | detachment/attachment | 1.87/0.66 | 2.28/0.79 | 1.65/0.69 |
| 6 | $C + C \rightleftharpoons C_2$ | attachment/detachment | 0.55/3.62 | 0.25/3.97 | 0.25/2.75 |
| 7 | $C + CH \rightleftharpoons C_2H$ | attachment/detachment | 0.48/2.95 | 0.49/3.19 | 1.27/2.95 |
| 8 | $CH + CH \rightleftharpoons C_2H_2$ | attachment/detachment | 0.29/2.21 | 0.24/2.28 | 0.14/2.28 |
| 9 | $C_2 + H \rightleftharpoons C_2H$ | attachment/detachment | 0.83/1.45 | 0.88/1.61 | 0.72/1.38 |
| 10 | $C_2H + H \rightleftharpoons C_2H_2$ | attachment/detachment | 1.01/1.67 | 1.07/1.68 | 0.85/1.64 |
| 11 | $C_2H_2$ | desorption | 1.59 | 0.89 | 1.83 |
| 12 | $C$ | diffusion | 0.15 | 0.11 | 0.50 |
| 13 | $CH$ | diffusion | 0.15 | 0.15 | 0.15 |
| 14 | $CH_2$ | diffusion | 0.14 | 0.13 | 0.18 |
| 15 | $C_2$ | diffusion | 0.48 | 0.58 | 0.49 |
| 16 | $C_2H$ | diffusion | 0.31 | 0.15 | 0.32 |
| 17 | $C_2H_2$ | diffusion | 0.36 | 0.30 | 0.44 |
| 18 | $H$ | diffusion | 0.14 | 0.13 | 0.13 |
| 19 | C+edge $\rightleftharpoons$ edge−C | edge attachment/detachment | 0.44/1.21 | 0.57/1.76 | 1.27/1.57 |
| 20 | CH+edge $\rightleftharpoons$ edge−CH | edge attachment/detachment | 0.33/0.83 | 0.61/1.25 | 0.44/1.08 |
| 21 | $CH_2$+edge $\rightleftharpoons$ edge−$CH_2$ | edge attachment/detachment | 0.06/1.79 | 0.46/2.35 | 0.19/2.08 |
| 22 | $C_2$+edge $\rightleftharpoons$ edge−$C_2$ | edge attachment/detachment | 1.22/2.24 | 1.21/2.31 | 0.58/2.19 |
| 23 | $C_2H$+edge $\rightleftharpoons$ edge−$C_2H$ | edge attachment/detachment | 0.9/1.74 | 0.82/1.96 | 0.56/2.31 |
| 24 | $C_2H_2$+edge $\rightleftharpoons$ edge−$C_2H_2$ | edge attachment/detachment | 0.93/1.72 | 0.85/1.85 | 1.05/- |
| 25 | H + edge $\rightleftharpoons$ edge−H | edge attachment/detachment | 0.77/1.61 | 0.81/1.79 | 0.64/1.53 |
| 26 | edge−CH $\rightleftharpoons$ edge−C+H | (de)hydrogenation | 1.76/0.71 | 1.5/0.54 | -/- |
| 27 | edge−$CH_2$ $\rightleftharpoons$ edge−CH+H | (de)hydrogenation | 2.36/0.59 | 2.74/0.93 | 2.04/0.73 |
| 28 | edge−$C_2H$ $\rightleftharpoons$ edge−$C_2$+H | (de)hydrogenation | 1.57/1.08 | 1.73/1.19 | 1.39/0.83 |
| 29 | edge−$C_2H_2$ $\rightleftharpoons$ edge−$C_2H$+H | (de)hydrogenation | 1.7/1.07 | 2.07/1.18 | -/- |

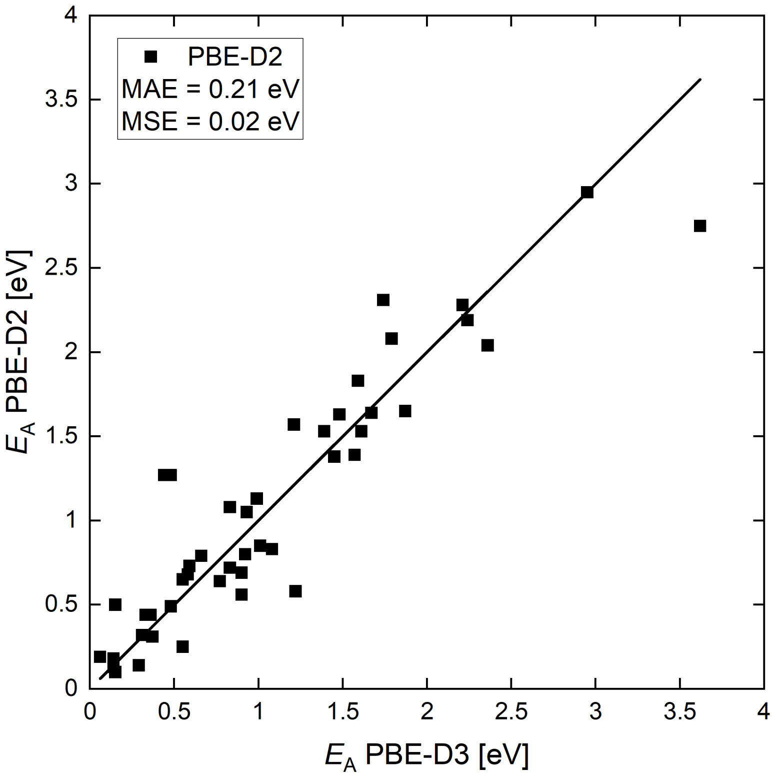


**Figure S3.** Parity plot comparing the energy barriers calculated with PBE-D3 functional (this work) vs. PBE-D2 functional [1].

**S2. Linear scaling relations**

Brøndsted, Evans and Polanyi showed that the activation energy scales linearly with the reaction energy, $E_{FS}$ [2]. This relation was already used in the homogeneous catalysis [3–5]. Nowadays, it is also widely used in heterogeneous catalysis[6–9], because it allows to calculate the activation energy barrier without explicitly calculating the transition state. Usually, calculating ground states and, therefore, reaction energies is straightforward, while the calculation of transition states is rather difficult and can also fail for very complex reactions. Here, we have employed the transition state scaling relations [10] to show that scaling relations for such reactions exist. Instead of the activation energy, like in the Brøndsted-Evans-Polanyi relation, the transition state energy, $E_{TS}$, was used. The reaction energy and the transition state energies of reaction:

$$xCH_{4(g)} + Cu \rightleftharpoons C_xH_{y\,(ads)} + \left(2x - \frac{y}{2}\right)H_{2\,(g)}, \tag{S1}$$

were calculated as:

$$\Delta E_{FS/TS} = E_{FS/TS} + \left(2x - \frac{y}{2}\right)E_{H_2} - E_{Cu} - xE_{CH_4}. \tag{S2}$$

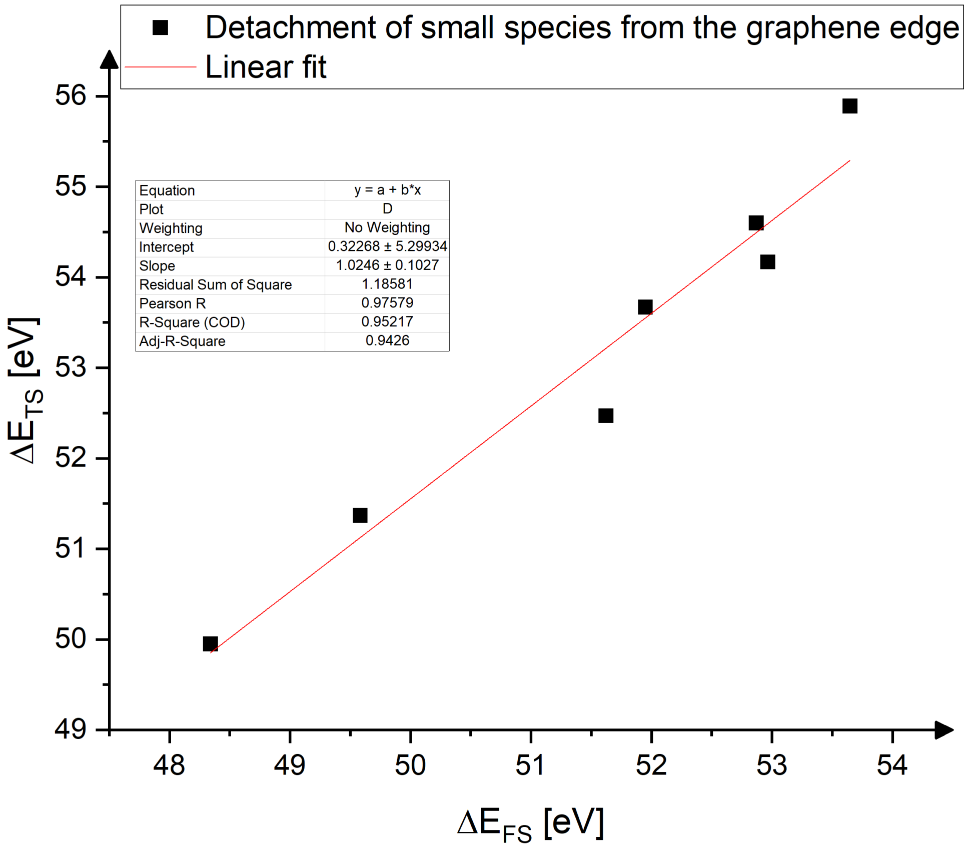


**Figure S4.** Reaction energies, $\Delta E_{FS}$, of small species: $H, C, C_2, CH, CH_2, C_2H$ and $C_2H_2$, attached to the graphene edge, plotted against the transition state energies, $\Delta E_{TS}$. A linear fit indicates the linear scaling relation.

First, we investigated the detachment of small species from the graphene edge (reactions 19-25 in Table S1). Here, the reaction energy was calculated as for the case where the small species is attached to the graphene edge. A similar procedure was also employed for the dehydrogenation reactions from the graphene edges (reactions 25-29 in Table S1). The reaction energy was calculated for the hydrogenated graphene edge (edge-H) respectively the hydrogenated small species attached to the graphene edge (i.e. edge-CH). In Figure S4 and Figure S5, the reaction energies are plotted against the transition state energies to demonstrate the scaling. The energy scale of the observed reaction energies and transition state energies is high, i.e. around 50 eV. It is connected to the fact that the formation of C on the Cu(111) surface from $CH_4$ costs roughly 4 eV. For the formation of the graphene edge we needed to arrange 36 carbon atoms in the graphene structure. Since graphene is much more stable than 36 carbon atoms on the surface, a total reaction energy is lower, however the reaction is still endothermic.

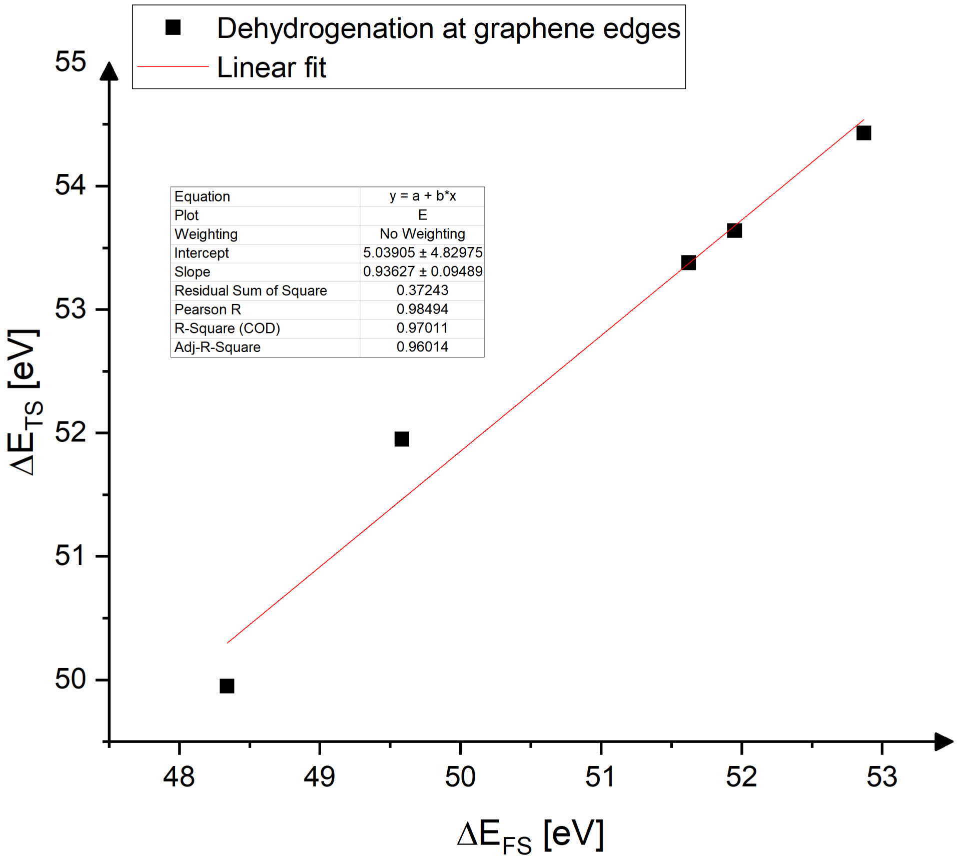


**Figure S5.** Reaction energies, $\Delta E_{FS}$, of the hydrogenated species: edge-H, edge-CH, edge-$CH_2$, edge-$C_2H$ and edge-$C_2H_2$, at the graphene edge against the transition state energies, $\Delta E_{TS}$. A linear fit indicates the linear scaling relation.

As it can be seen, in both figures the transition state energy scales linearly with the reaction energy. The observed Pearson correlation coefficient, which measures the linear correlation between two sets of data, is $> 0.97$. Therefore, it should be possible to estimate activation energy barriers for similar reactions.

## S3. KMC calculations

### *S3.1. Lattice construction and rates used in KMC simulations*

We mapped the Cu(111) surface into the honeycomb lattice shown in Figure S6. It contains fcc and hcp adsorption sites where each site has three neighbors of the other type. The neighboring distance and lattice vectors are 0.142 nm, and 0.246 nm, respectively. With this consideration, honeycomb lattices of different sizes could be generated. The one we chose to perform simulations is on a $100 \times 100$ nm$^2$ lattice with 388206 points. We defined the neighboring rule in a way to fill the neighborhood list of each point with three other points. Then, for points in a border that do not have 3 neighbors, we considered the missing spots as the mirror side of the lattice. However, because the simulation begins with a ribbon from the left as the first flake, it is not practical to have PBC from both the left and right sides.

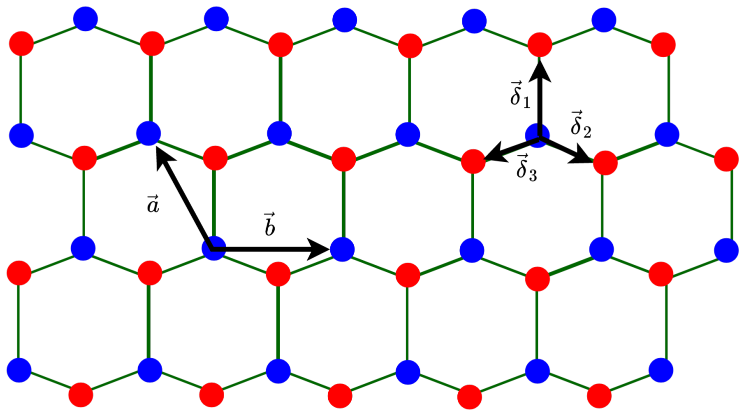


**Figure S6.** Illustration of the honeycomb lattice formed via fcc and hcp sub-lattices in blue and red. $\vec{a}$ and $\vec{b}$ are lattice vectors and the nearest neighbor distances are $\vec{\delta}_i$ (i=1, 2, 3).

With a fixed temperature of 1300 K, we calculated all reaction rates, except dissociative adsorption of methane and hydrogen, using the following equation:

$$r_T = \frac{K_B T}{h} exp(-\frac{E_a}{K_B T}) \tag{S3}$$

where $K_B$, $h$, and $E_a$ are Boltzmann constant, Planck constant, activation energy barrier, respectively, and $K_B h/T$ is the pre-exponential factor. We calculated $H_2$ adsorption (shown in Table 4 in the main body) based on the approach introduced in Ref. [1] as:

$$r^{ad}_{H_2} = \frac{h^2}{(2\pi m_{H_2} K_B T)^{3/2}} \frac{P_{H_2} q^{vib}_{TS,H_2}}{q^{vib}_{gas,H_2} q^{rot}_{gas,H_2}} exp(-\frac{E_a}{K_B T}), \tag{S4}$$

where

- $q^{vib}_{TS,H_2} = 54.96$, is $H_2$ vibrational partition function in the transition state at $T = 1300$.
- $q^{vib}_{gas,H_2} = 1.009$, is the gas phase $H_2$ vibrational partition function at $T = 1300$.
- $q^{rot}_{gas,H_2} = 7.466$, is the gas phase $H_2$ rotational partition function at $T = 1300$.
- $P_{H_2}$ is the partial pressure of $H_2$.
- $m_{H_2}$ is the mass of $H_2$.

we did the same for methane adsorption (shown in Table 4) as:

$$r^{ad}_{CH_4} = \frac{h^2}{(2\pi m_{CH_4} K_B T)^{3/2}} \frac{P_{CH_4} q^{vib}_{TS,CH_4}}{q^{vib}_{gas,CH_4} q^{rot}_{gas,CH_4}} exp(-\frac{E_a}{K_B T}), \tag{S5}$$

where

- $q^{vib}_{TS,CH_4} = 64532.53$, is $CH_4$ vibrational partition function in the transition state at $T = 1300$.
- $q^{vib}_{gas,CH_4} = 3.575$, is the gas phase $CH_4$ vibrational partition function at $T = 1300$.
- $q^{rot}_{gas,CH_4} = 352.13$, is the gas phase $CH_4$ rotational partition function at $T = 1300$.
- $P_{CH_4}$ is the partial pressure of $CH_4$.
- $m_{CH_4}$ is the mass of $CH_4$.

For the whole lattice these adsorption rates were calculated via $N_{free} \times r^{ad}$, where $N_{free}$ is the number of available surface sites.

Table S2 includes 55 reactions ranging from dissociative adsorption of gases, attachment of different species to edge, (de)hydrogenation of attached species, and ring closures, their barriers and rates used in KMC simulations. We calculated all barriers, except the ring formation and $C_2$ flipping at edge (they were taken from Chen et al [11]), using our DFT

approach (with PBE-D3 functional). Rates of reactions 1 to 29 are the same as reactions 1 - 29 in Table S1 for the PBE-D3 functional.

**Table S2.** Reactions considered in the KMC simulation.

| Entry | Reaction | Type | Barrier (eV) | Rate ($s^{-1}$) |
|---|---|---|---|---|
| 1 | $H_2 \rightleftharpoons H+H$ | adsorption/desorption | 0.37/0.92 | –/7.35×10$^9$ |
| 2 | $CH_4 \rightleftharpoons CH_3+H$ | adsorption/desorption | 1.48/0.90 | –/8.78×10$^9$ |
| 3 | $CH_3 \rightleftharpoons CH_2+H$ | detachment/attachment | 1.39/0.58 | 1.11×10$^8$ /1.53×10$^{11}$ |
| 4 | $CH_2 \rightleftharpoons CH+H$ | detachment/attachment | 0.99/0.55 | 3.93× 10$^9$ /1.99×10$^{11}$ |
| 5 | $CH \rightleftharpoons C + H$ | detachment/attachment | 1.87/0.66 | 1.52× 10$^6$ /7.48×10$^{10}$ |
| 6 | $C + C \rightleftharpoons C_2$ | attachment/detachment | 0.55/3.62 | 1.99×10$^{11}$ /2.51×10$^{-1}$ |
| 7 | $C + CH \rightleftharpoons C_2H$ | attachment/detachment | 0.48/2.95 | 3.73× 10$^{11}$ /9.92×10$^1$ |
| 8 | $CH + CH \rightleftharpoons C_2H_2$ | attachment/detachment | 0.29/2.21 | 2.03× 10$^{12}$ /7.33×10$^4$ |
| 9 | $C_2 + H \rightleftharpoons C_2H$ | attachment/detachment | 0.83/1.45 | 1.64×10$^{10}$ /6.48×10$^7$ |
| 10 | $C_2H + H \rightleftharpoons C_2H_2$ | attachment/detachment | 1.01/1.67 | 3.29×10$^9$ /9.09×10$^6$ |
| 11 | $C_2H_2$ | desorption | 1.59 | 1.86×10$^7$ |
| 12 | C | diffusion | 0.15 | 7.1× 10$^{12}$ |
| 13 | CH | diffusion | 0.15 | 7.1× 10$^{12}$ |
| 14 | $CH_2$ | diffusion | 0.14 | 7.76×10$^{12}$ |
| 15 | $C_2$ | diffusion | 0.48 | 3.73×10$^{11}$ |
| 16 | $C_2H$ | diffusion | 0.31 | 1.07×10$^{12}$ |
| 17 | $C_2H_2$ | diffusion | 0.36 | 1.09×10$^{12}$ |
| 18 | H | diffusion | 0.14 | 7.76× 10$^{12}$ |
| 19 | $C+edge \rightleftharpoons edge-C$ | edge attachment/detachment | 0.44/1.21 | 5.33×10$^{11}$ /5.52×10$^8$ |
| 20 | $CH+edge \rightleftharpoons edge-CH$ | edge attachment/detachment | 0.33/0.83 | 1.42×10$^{12}$ /1.64×10$^{10}$ |
| 21 | $CH_2+edge \rightleftharpoons edge-CH_2$ | edge attachment/detachment | 0.06/1.79 | 1.58×10$^{13}$ /3.11×10$^6$ |
| 22 | $C_2+edge \rightleftharpoons edge-C_2$ | edge attachment/detachment | 1.22/2.24 | 5.05×10$^8$ /5.06×10$^4$ |
| 23 | $C_2H+edge \rightleftharpoons edge-C_2H$ | edge attachment/detachment | 0.9/1.74 | 8.78×10$^9$ /4.87×10$^6$ |
| 24 | $C_2H_2+edge \rightleftharpoons edge-C_2H_2$ | edge attachment/detachment | 0.93/1.72 | 6.72×10$^9$ /5.81×10$^6$ |
| 25 | $H + edge \rightleftharpoons edge-H$ | edge attachment/detachment | 0.77/1.61 | 2.80×10$^{10}$ /1.55×10$^7$ |
| 26 | $edge-CH \rightleftharpoons edge-C+H$ | (de)hydrogenation | 1.76/0.71 | 4.07×10$^6$ /4.79×10$^{10}$ |
| 27 | $edge-CH_2 \rightleftharpoons edge-CH+H$ | (de)hydrogenation | 2.36/0.59 | 1.92×10$^4$ /1.39×10$^{11}$ |
| 28 | $edge-C_2H \rightleftharpoons edge-C_2+H$ | (de)hydrogenation | 1.57/1.08 | 2.22×10$^7$ /1.76×10$^9$ |
| 29 | $edge-C_2H_2 \rightleftharpoons edge-C_2H+H$ | (de)hydrogenation | 1.7/1.07 | 6.95×10$^6$ /1.93×10$^9$ |
| 30 | C | Ring (de)formation | 2.29/0.54 | 3.59×10$^4$ /2.18×10$^{11}$ |
| 31 | $C_2$ | Ring (de)formation | 2.29/0.54 | 3.59×10$^4$ /2.18×10$^{11}$ |
| 32 | $C_2$ | Rotation at edge | 0.74 | 3.66×10$^{10}$ |

**Note**: Reaction rates of $CH_4$ and $H_2$ gas adsorption depend on their pressure and the number of available sites on the lattice (see Table 4 in the main body).

### *S3.2. The steady state time and concentration*

As it is shown in Figures S7 and S8, about $2 \times 10^{-3}$ s is required for the system to reach steady state, where the concentration of species fluctuates around a converged value of their moving average[12].

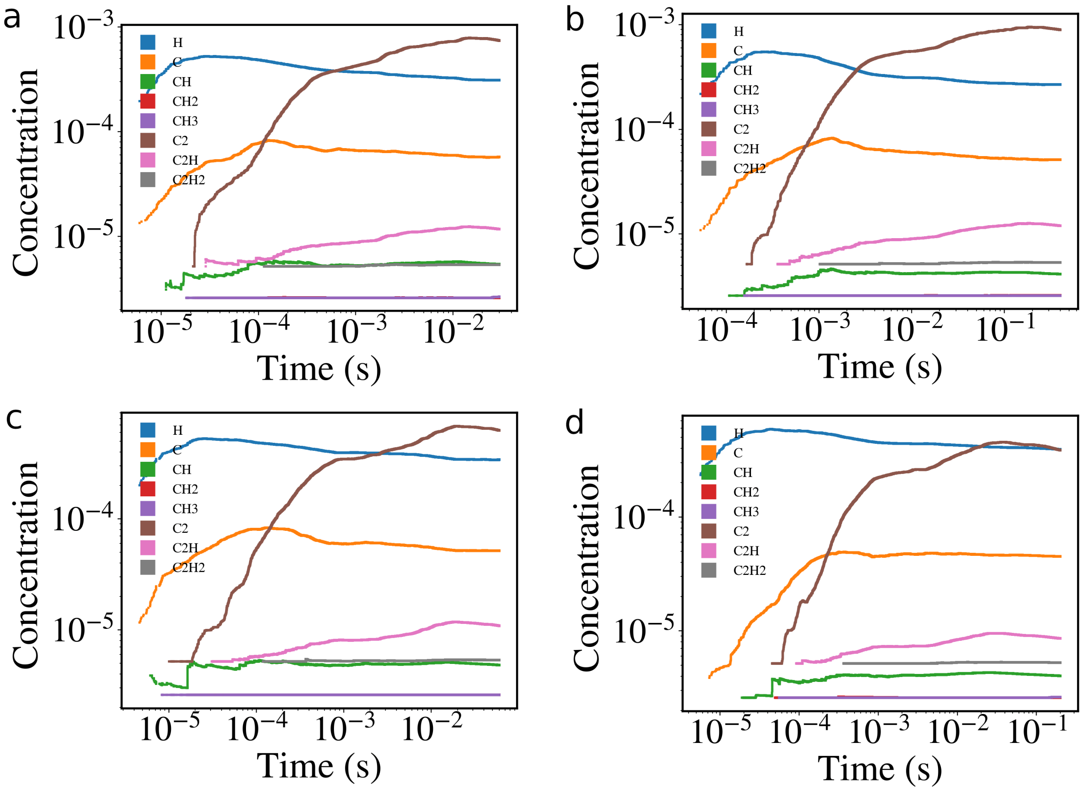


**Figure S7.** Evolution of the moving average concentration of different species as a function of time for samples S1 (a), S2 (b), S3 (c), and S4 (d) (see Table 1).

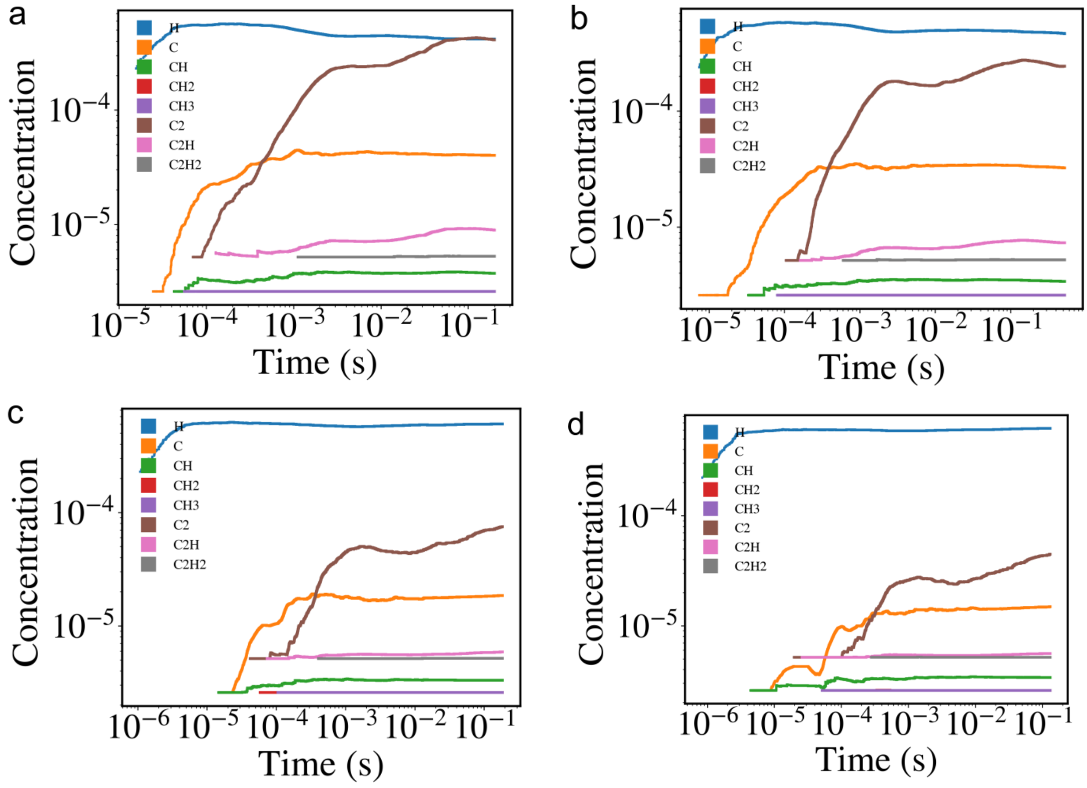


**Figure S8.** Evolution of the moving average concentration of different species as a function of time for samples S5 (a), S6 (b), S7 c), and S8 (d) (see Table 1).

Steady state concentration of species for all simulations are shown in Figure S9. The influence of partial pressures on the concentration of species are discussed in the main text.

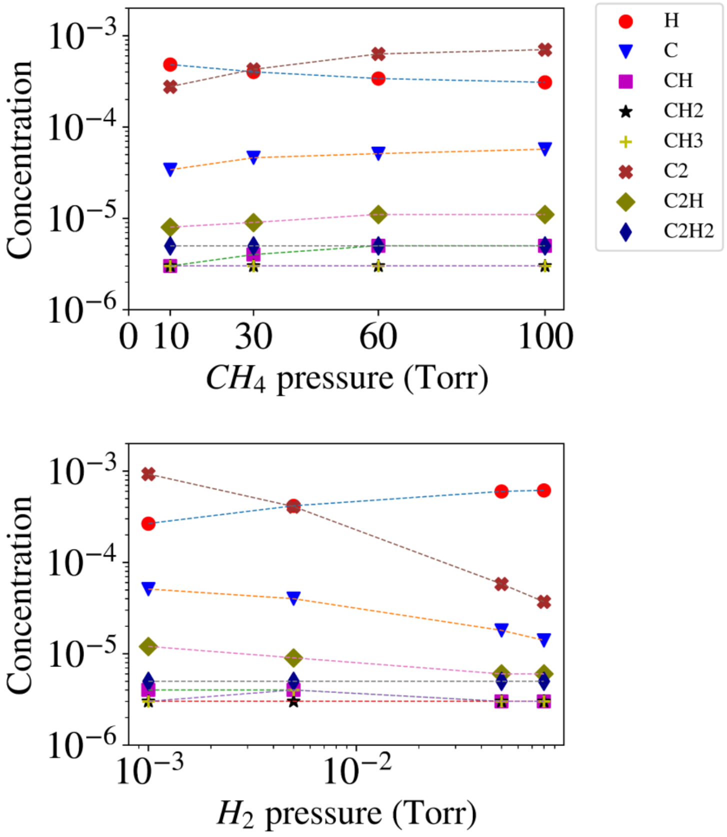


**Figure S9.** Species concentration profile for different $CH_4$ and $H_2$ partial pressures.

Since we discussed the spatio-temporal evolution of graphene growth for the sample S1 in the main text, we provide the same analyses for other samples considered in this study (see Table 1 in the main body) in Figures S10 to S16. We realized that for the samples with the higher methane partial pressure, less time is needed to reach the similar flake radius. For others, longer simulation time was required (see Table S3).

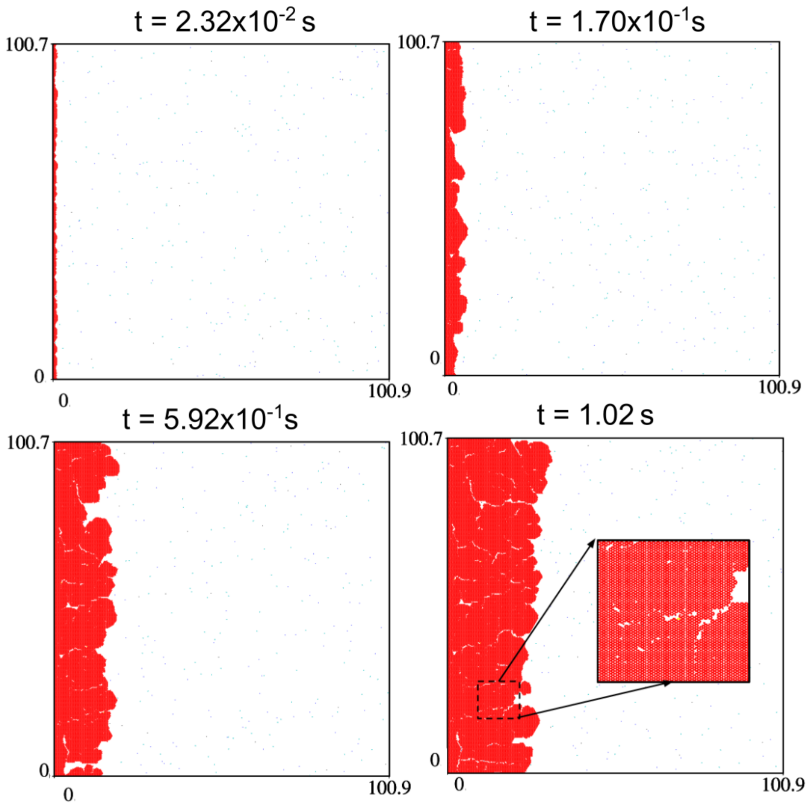


**Figure S10.** Spatio-temporal evolution of graphene growth for $P_{CH_4}$ = 10 and $P_{H_2}$ = 0.001 Torr (sample S2).

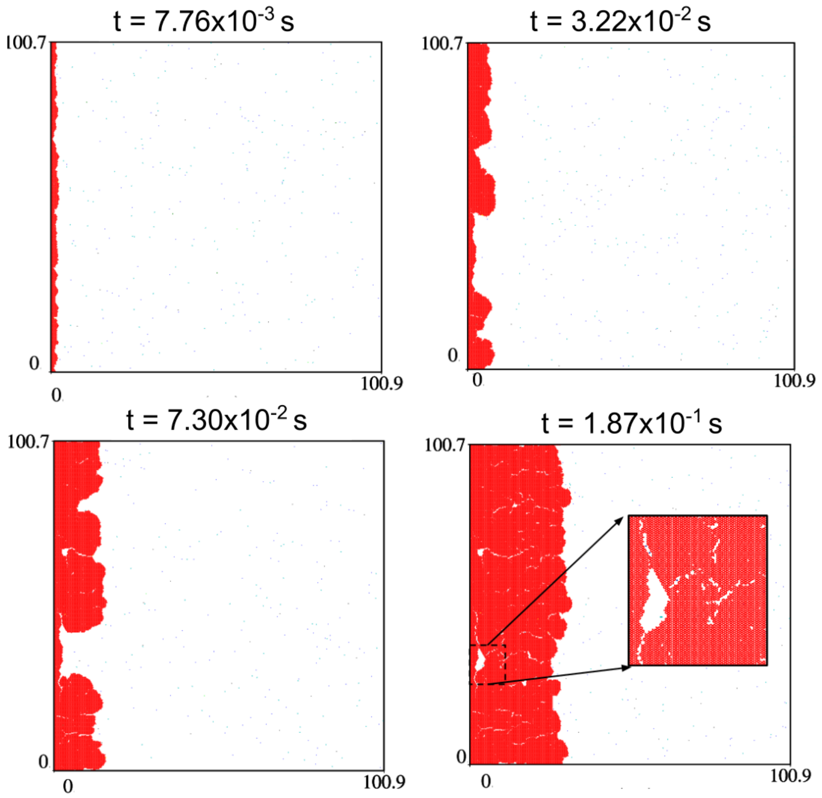


**Figure S11.** Spatio-temporal evolution of graphene growth for $P_{CH_4}$ = 60 and $P_{H_2}$ = 0.01 Torr (sample S3).

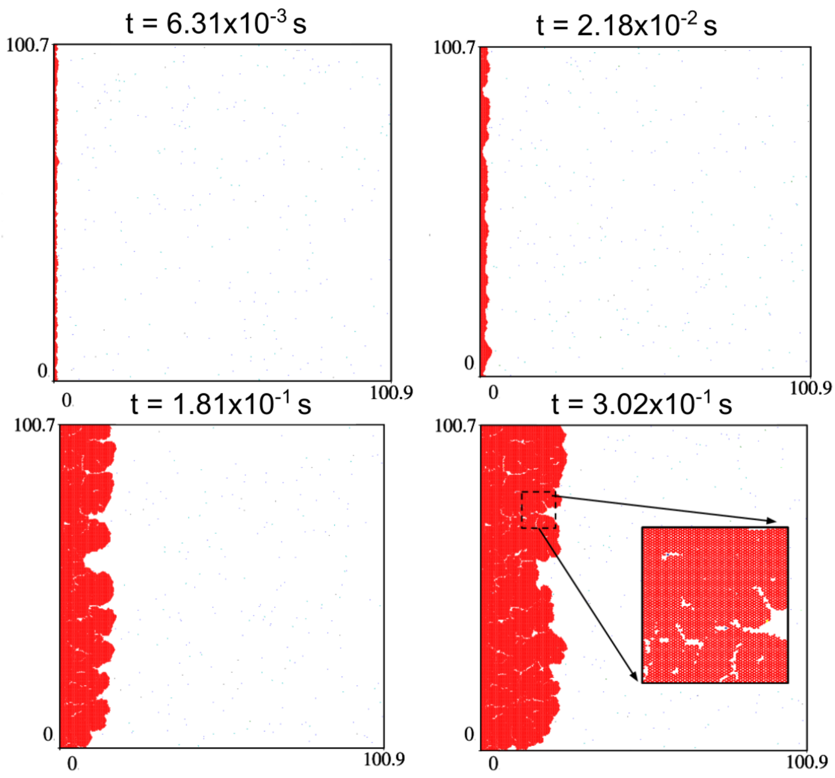


**Figure S12.** Spatio-temporal evolution of graphene growth for $P_{CH_4}$ = 30 and $P_{H_2}$ = 0.01 Torr (sample S4).

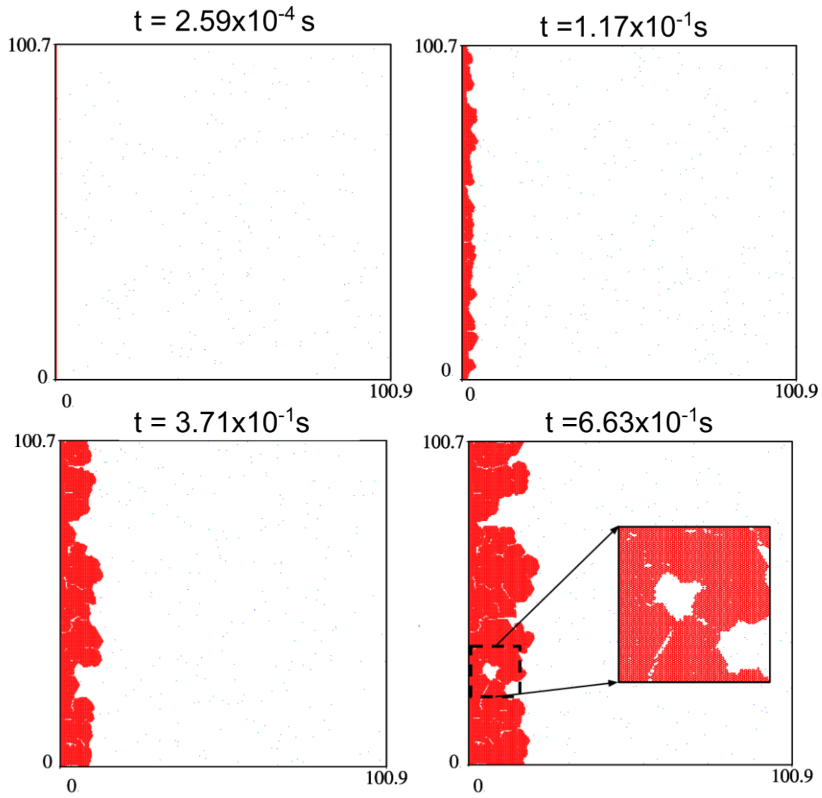


**Figure S13.** Spatio-temporal evolution of graphene growth for $P_{CH_4}$ =10 and $P_{H_2}$ = 0.005 Torr (sample S5).

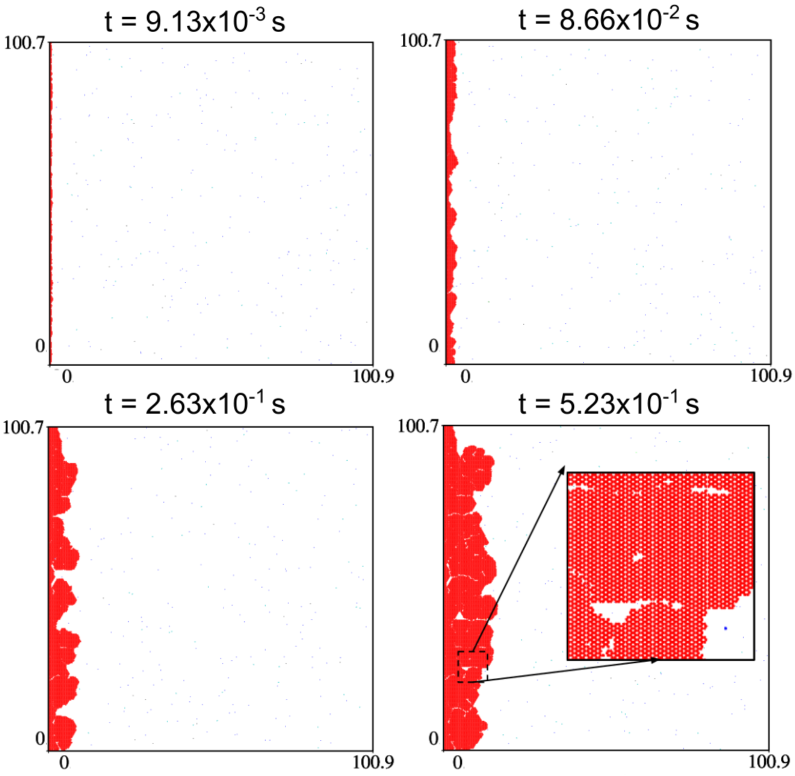


**Figure S14.** Spatio-temporal evolution of graphene growth for $P_{CH_4}$ = 10 and $P_{H_2}$ = 0.01 Torr (sample S6).

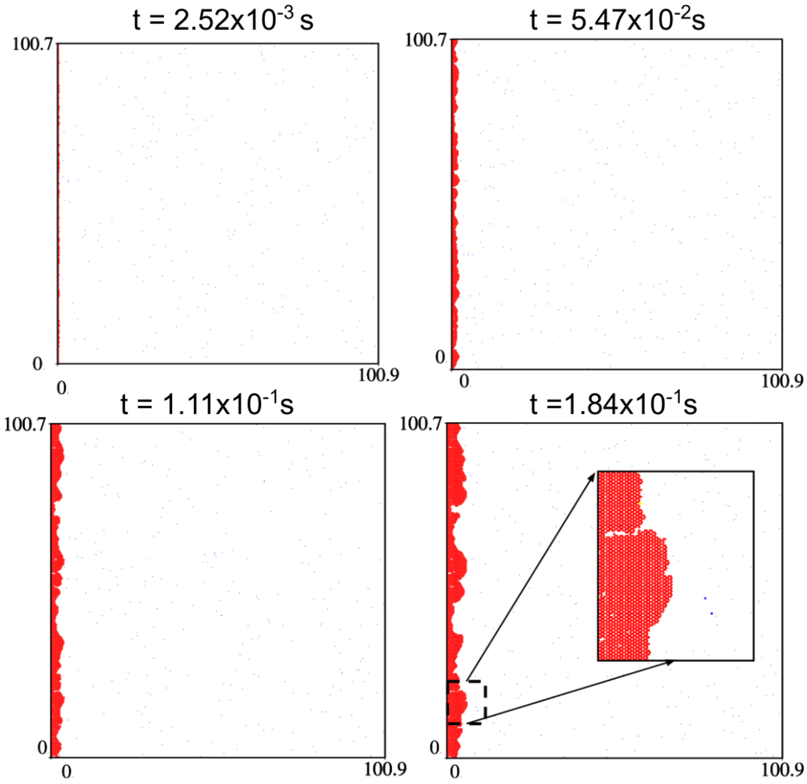


**Figure S15.** Spatio-temporal evolution of graphene growth for $P_{CH_4}$ = 10 and $P_{H_2}$ = 0.05 Torr (sample S7).

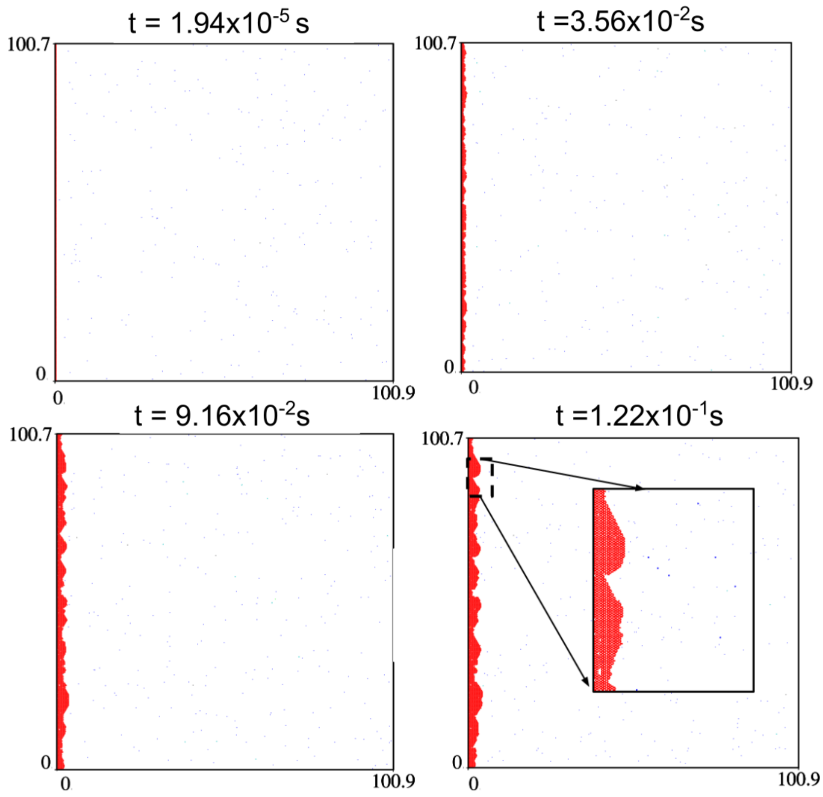


**Figure S16.** Spatio-temporal evolution of graphene growth for $P_{CH_4}$ = 10 and $P_{H_2}$ = 0.08 Torr (sample S8).

**Table S3.** Summary on the mean radius ($R_{mean}$) and simulated time for all samples.

| **Sample** | $P_{CH_4}$ **(Torr)** | $P_{H_2}$ **(Torr)** | $R_{mean}$ **(nm)** | **time (s)** |
|---|---|---|---|---|
| S1 | 100 | 0.01 | 38.23 | 0.157 |
| S2 | 10 | 0.001 | 25.95 | 1.018 |
| S3 | 60 | 0.01 | 30.53 | 0.2 |
| S4 | 30 | 0.01 | 22.87 | 0.302 |
| S5 | 10 | 0.005 | 17.29 | 0.709 |
| S6 | 10 | 0.01 | 12.40 | 0.523 |
| S7 | 10 | 0.05 | 3.85 | 0.184 |
| S8 | 10 | 0.08 | 2.86 | 0.131 |

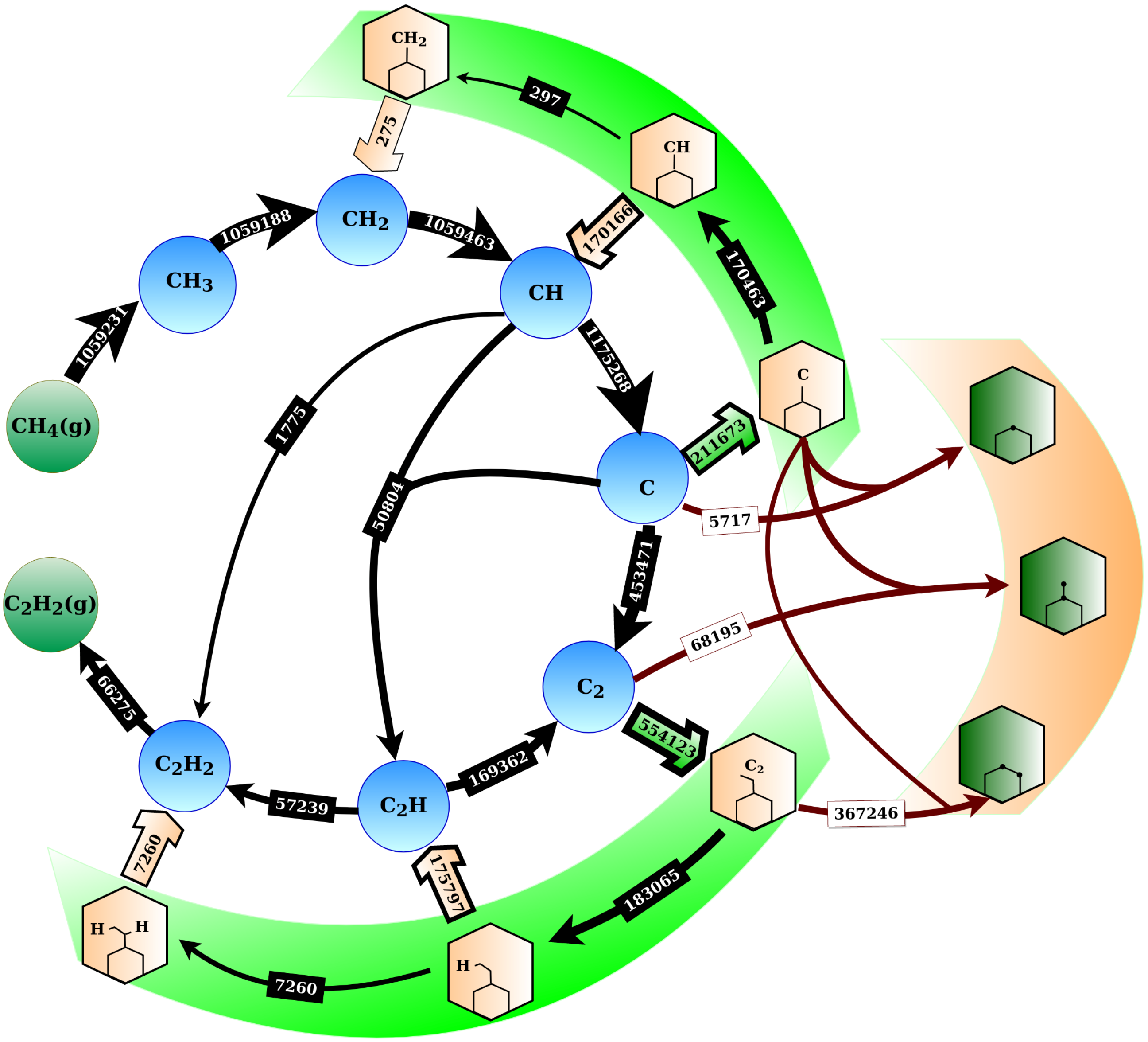


**Figure S17.** Occurrence map of elementary reactions involved in the graphene growth process on Cu(111) at $p(CH_4) = 100$ Torr, $p(H_2) = 0.01$ Torr, and $T = 1300$ K (sample S1, see Table 1). The map summarizes net contributions (per second) of the most relevant events in the KMC simulation for 0.138 s. Blue and green circles indicate free species on the lattice. Green highlighted regions represent attachment of species to the flake edges, while brown highlighted region shows the hexagon formation via ring closure reactions on the edges. $H_2$ dissociative adsorption and desorption, as well as diffusion of species, are not shown here. Gas phase species are marked with a "$g$". The possible conversions are shown as the arrows in the direction of the net contribution (forward minus backward occurrences) . The numbers on arrows are the net contribution per second.

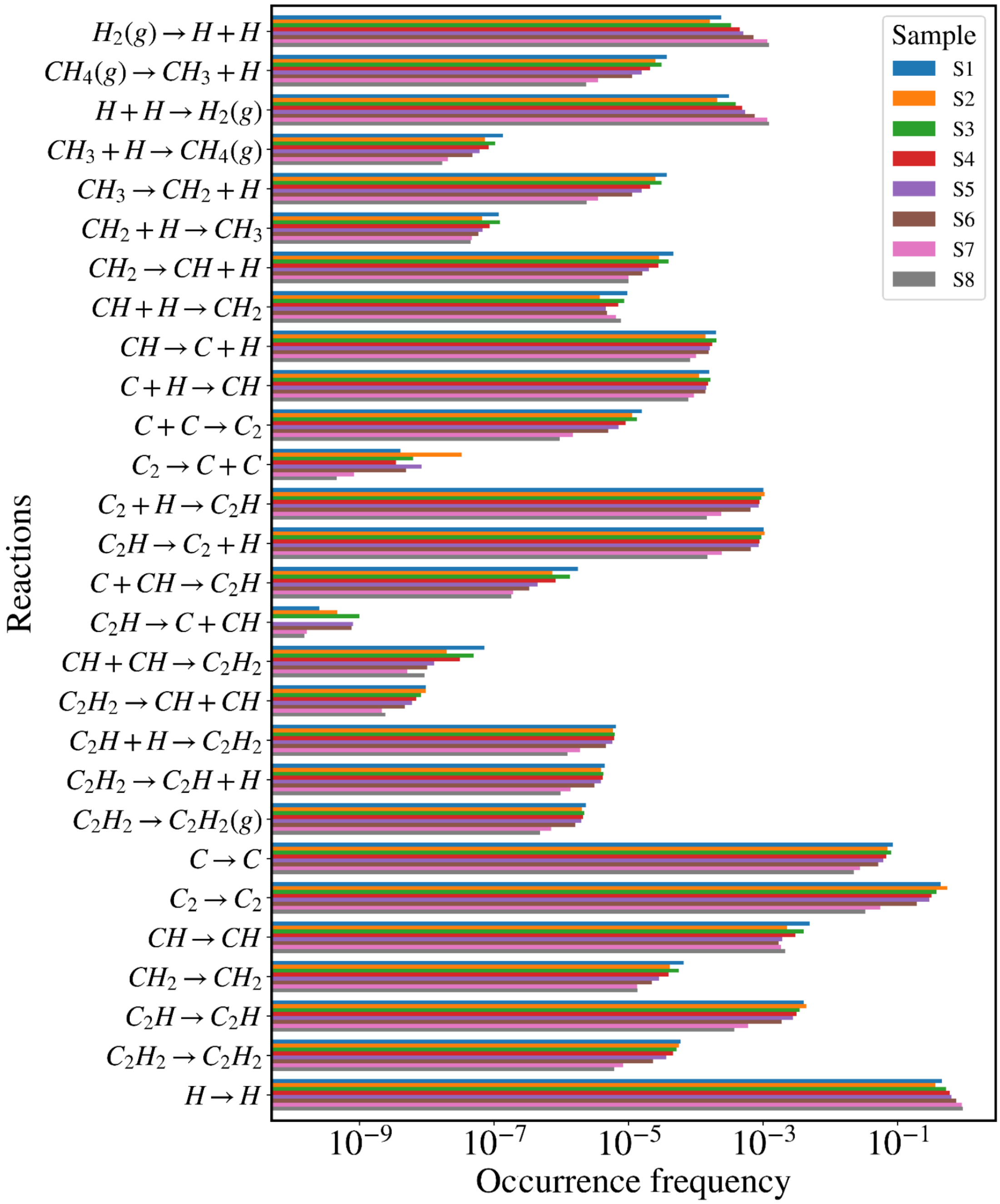


**Figure S18.** Occurrence frequency of a the first 28 reactions in Table S2 (i.e. reaction occurrence per the whole number of reactions) for all samples simulated (listed in Table 1 and Table S3).

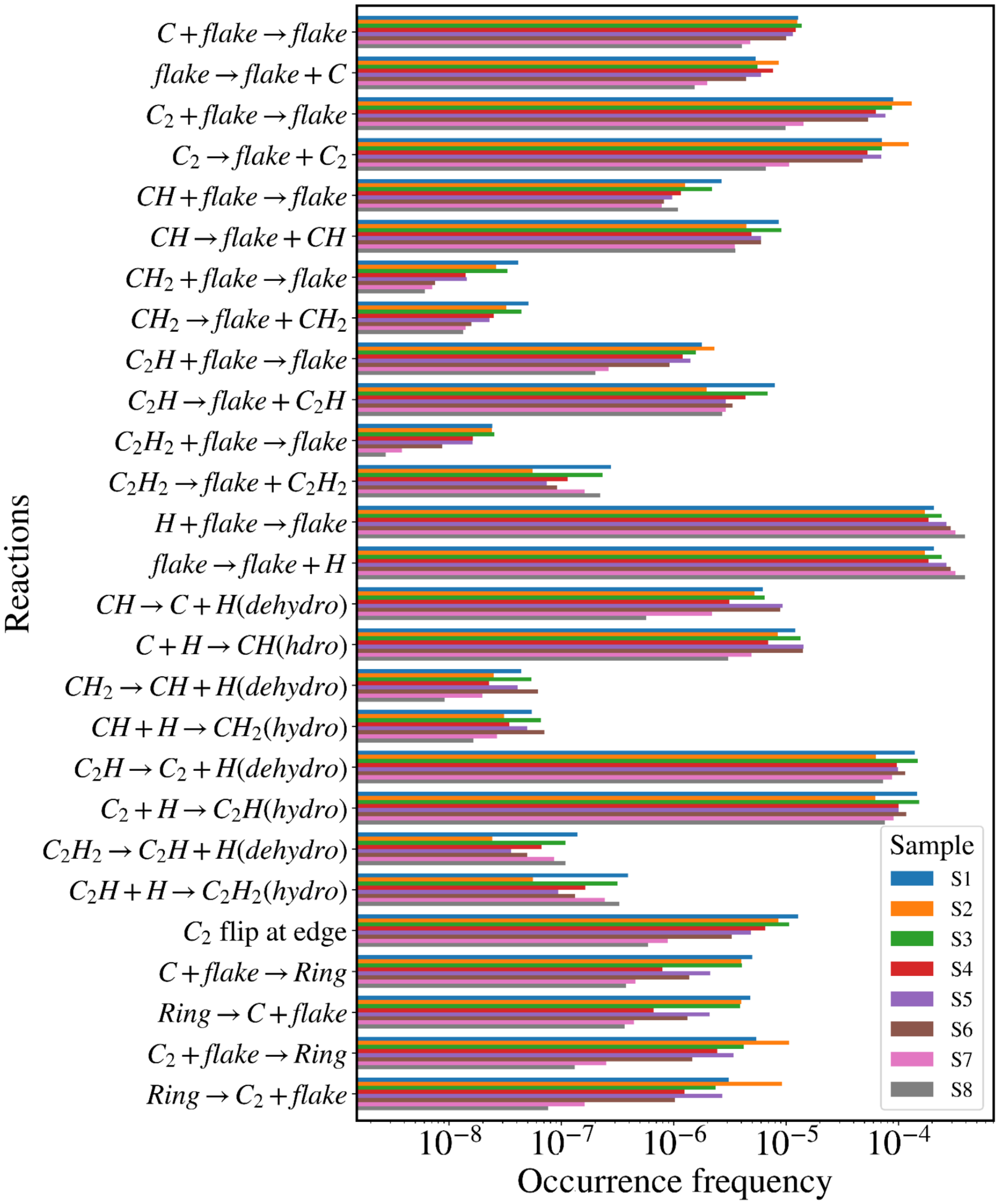


**Figure S19.** Occurrence frequency of reaction number 29 to 55 in Table S2 (i.e. reaction occurrence per the whole number of reactions) for all samples.

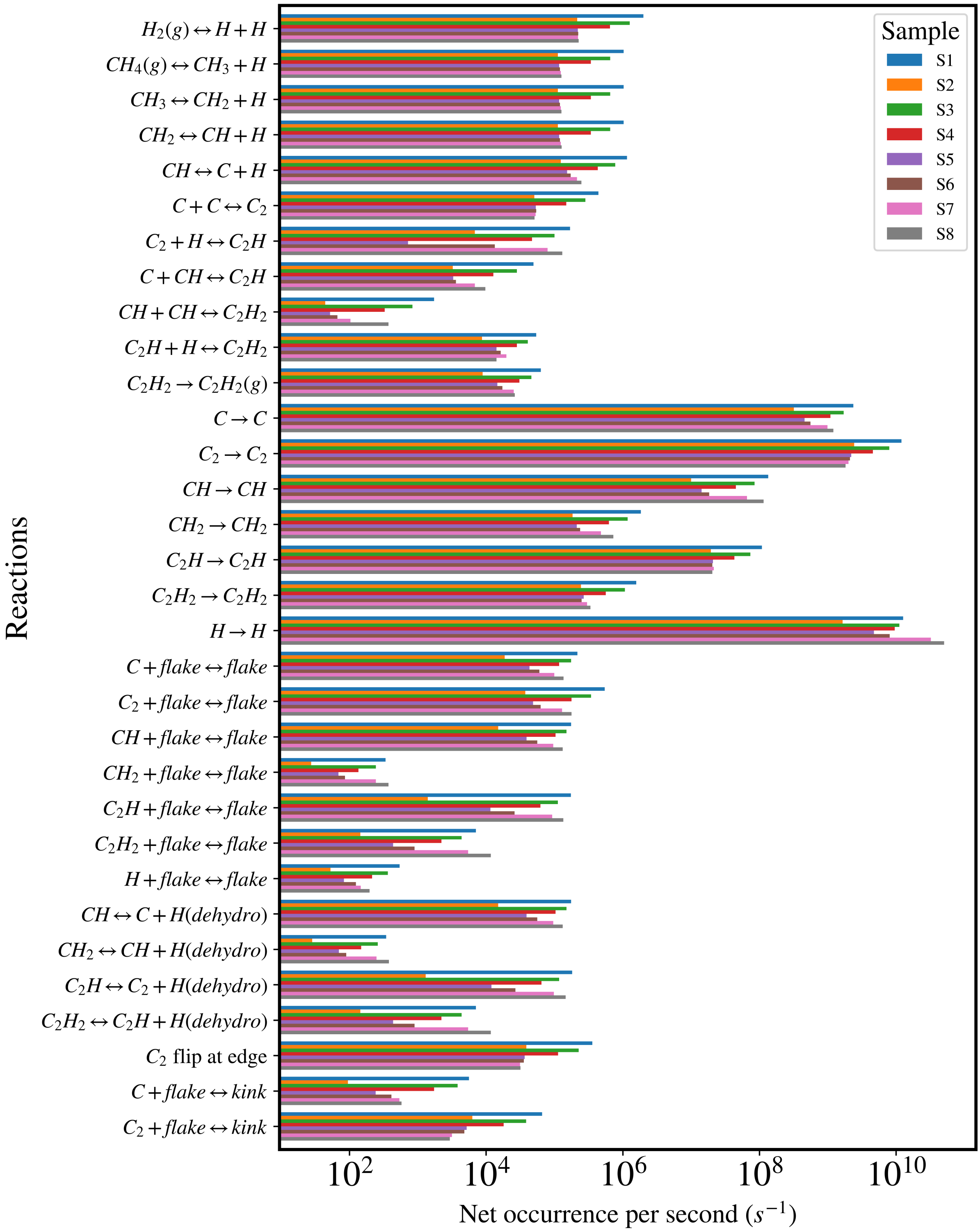


**Figure S20.** Net occurrence of reactions per second for all samples. Numbers are considered positive in the dominant direction of reactions.

*S3.3. Correlation of the growth rate and the ratio of partial pressure*

As we mentioned in the main text, increasing the $CH_4$ partial pressure leads to larger flakes s ize. This means there will be less free adsorption sites available with time that results in a decrease in the growth rate during graphene growth. In Figure S21 the time-dependence of the growth rate for methane partial pressure samples is shown.

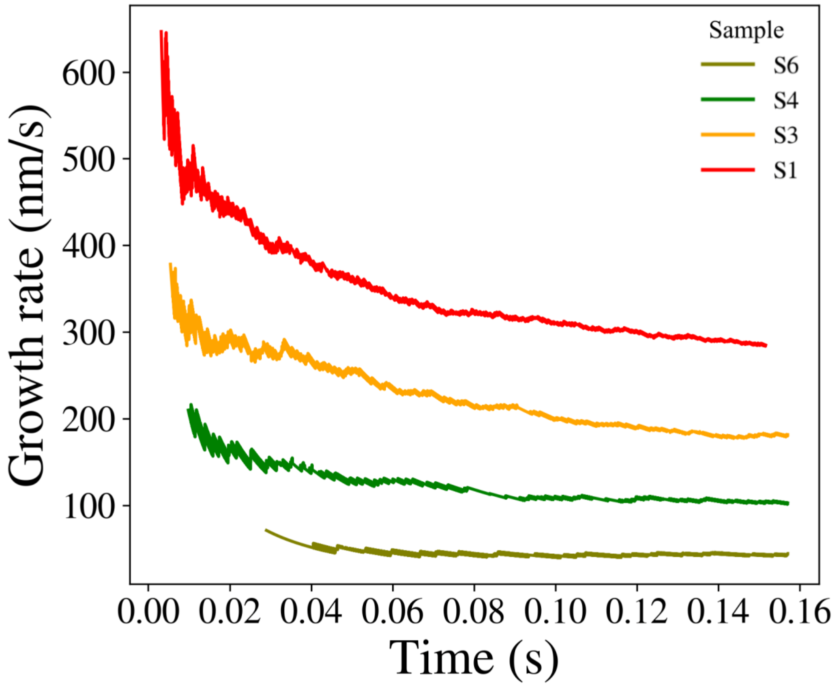


**Figure S21.** Time series of graphene growth rate for different $CH_4$ partial pressures. The change of the flake growth rate for samples S1 (100 Torr), S3 (60 Torr), S4 (30 Torr) and S6 (10 Torr) are shown with the red, orange, green and olive color, respectively.

For all samples calculated in KMC simulations, the ratio of partial pressures was defined as $R_p = P_{H_2}/P_{CH_4}$. Figure S22 shows the changes of growth rate and flake radius for all samples. We observed that for sample with low $R_p$, while the partial pressure of methane is high (samples S1, S3, S4), the radius and growth rate are higher. This means these samples required less time to reach the same flake size in comparison to the other samples.

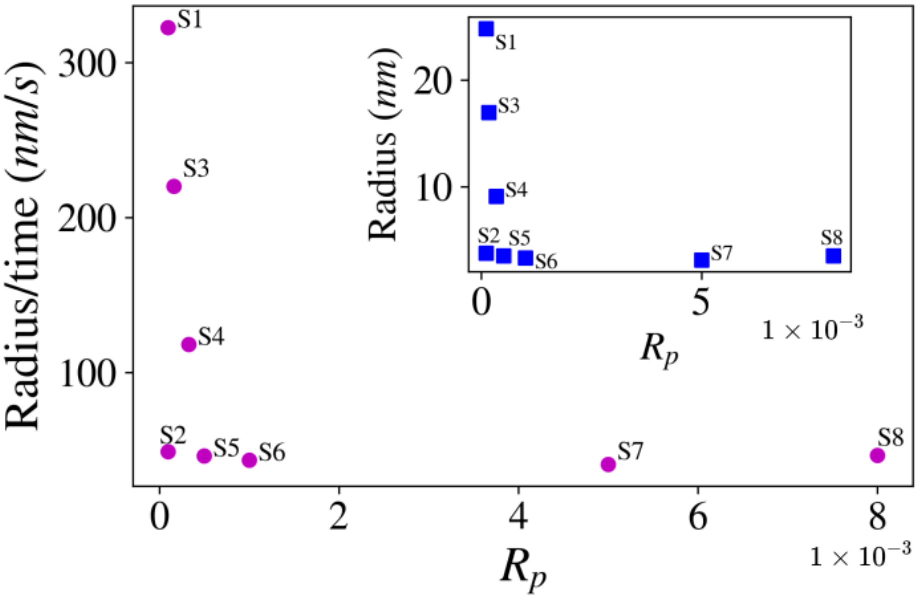


**Figure S22.** The correlation between the graphene growth rate and the ratio of the partial pressure of $CH_4$ and $H_2$. The correlation between the flake radius is given in the inset.

*S3.4. Surface roughness*

Considering the surface as a 100×100 nm$^2$ lattice (while graphene flake grows from the left side of the lattice), we defined the radius ($R_i$) as the distance of each outer edge point from the vertical axis at the left hand side (see Figure S23). Then, we calculated the mean radius ($R_{mean}$) as the average radius over the evaluation length (L) and defined the average roughness parameter ($R_a$) as the deviation of $R_i$ from the mean radius ($R_{mean}$) over the vertical axis (the evaluation length, (L)) as:

$$R_a = \frac{1}{L}\sum_{n=1}^{L}|R_i - R_{mean}|. \tag{S6}$$

The root mean square roughness ($R_q$) was defined as the root-mean-square of the deviation of $R_i$ from the mean radius ($R_{mean}$) over the evaluation length, (L) as:

$$R_q = \sqrt{\frac{1}{L}\sum_{n=1}^{L}(R_i - R_{mean})^2}. \tag{S7}$$

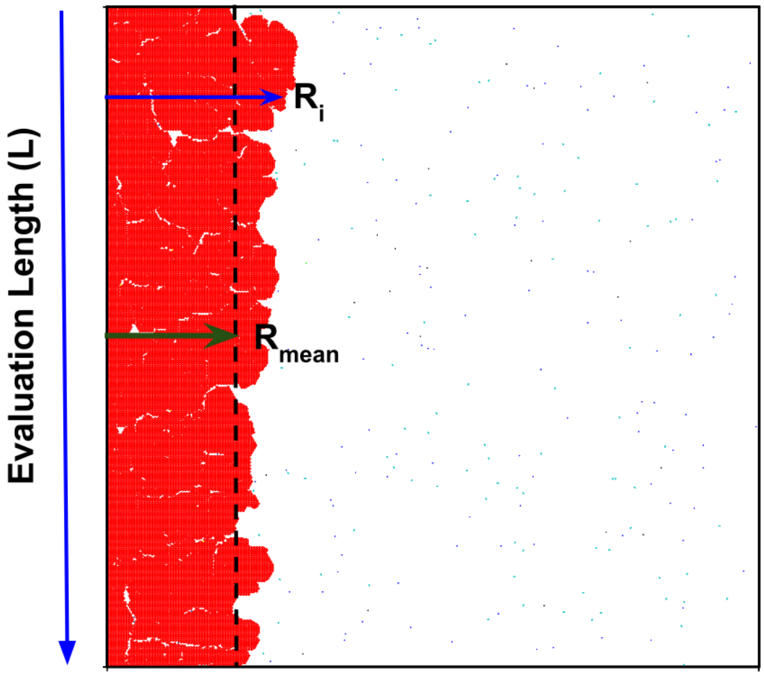


**Figure S23.** Schematic representation of the surface with a flake (in red), where the radius, $R_i$, mean radius $R_{mean}$, and evaluation length, L are shown.

## S4. Comparison with previous models

The model developed in this study uses the first principle calculations and KMC simulations to study graphene growth under different partial pressures of $CH_4$ and $H_2$ as precursors (sample from Table 1). We consider a list of 55 reactions representing reactions of carbon-containing species on graphene and ring closure reactions (see Table S2). All simulations performed on 100×100 nm$^2$ honeycomb lattice with 388206 sites at T=1300 K. The largest size of the graphene flake simulated (grown) is up to 40 nm (4293 Million simulation steps), obtained in 0.157 s for the sample S1 with $P_{CH_4}$=100, and $P_{H_2}$=0.01 Torr. Table S4 summarizes other DFT+KMC models developed for graphene growth in comparison to the multiscale model reported in the present study.

**Table S4.** The comparison of the present model with other DFT+KMC theoretical models for graphene growth.

| DFT details | KMC details | Simulated conditions | Graphene size and time | Other differences | Reference |
|---|---|---|---|---|---|
| PW-DFT[1], BEEF-vdW[2], PBE-D3[3], 450 eV, NEB[4] for transition state (TS), 3 × 3 and 10 × 3 slab, 55 reactions | Rejection-free KMC algorithm, random redistribution for surface diffusion reactions, 100×100 $nm^2$ honeycomb lattice with 388206 adsorption sites | 1300 K, 10-100 Torr and $10^{-3}$-$8\times10^{-2}$ Torr as partial pressures of $CH_4$ and $H_2$, initial flake as a ribbon at left, $CH_4$ and $C_xH_x$ for deposition | Up to 40 nm graphene flake at 0.157 s simulation time | Includes: subsurface processes, ring closure reactions, (de)hydrogenation reactions, no nucleation | Present work |
| PW-DFT, PBE-D2[5], 400 eV, climbing image NEB for TS, 4-layer slab, 44 reactions | Rejection-free KMC simulation, MFA[6] for hydrogenation and dehydrogenation reactions (free species), rectangular 500×500 lattice | 1300 K, an initial 200×200 size graphene flake, 1-$10^3$ Torr and $10^{-3}$-1 Torr as partial pressures of $CH_4$ and $H_2$ | Not available | No ring closure reactions, lattice with specific neighboring rule, study the effects of $C_3H_x$, edge structure evolution, and local environment | Ref.[1] |
| PW-DFT, PBE (dispersion correction is not available), 400 eV, climbing image NEB for TS, 4-layer slab, 28 reactions | Gillespie algorithm, hexagonal lattice with 20000 sites (213×246 Å$^2$) | 870 - 1273 K, an initial circular nucleus of graphene domain (diameter of 4 nm), deposition of carbon atoms as the growth source, deposition flux 0.1-10 $MLs^{-1}$ | Flakes sizes up to 90% coverage of the simulation box (213×246 Å$^2$), up to 4 seconds simulation time, different graphene mythologies: hexagonal, fractal (incl. six-fold symmetry shapes) and circular | No nucleation, limited list of reactions consists of carbon and its dimer reactions, morphology evolution of flakes based on the carbon deposition flux and temperature | Ref.[11] |
| PW-DFT, PBE (dispersion correction is not available), 420 eV, 9-monolayer substrate, activation energies of carbon diffusion events on Cu(111), 12 reactions | Rejection-free KMC algorithm adopted from NASCAM code[13], honeycomb lattice with 12800 sites | 1273 K, deposition of carbon atoms as the growth source, deposition flux $10^{-3}$-$10^5$ $MLs^{-1}$ | Flakes coverage up to 80% of the simulation box, up to 15 s simulation time | Limited list of reactions due to considering reactions of C monomer and its dimer, effects of the deposition flux on island edges and flakes morphology | Ref.[14] |
| PW-DFT, PBE-D2, $E_{cutoff}$ is not available, 3-layers substrate, 14 reactions | Rejection free KMC algorithm, hexagonal lattice | An initial graphene flake made of hexagons, simulation time in unit of $t_0 = h/K_BT$ | Up to 10 $\mu$m flake sizes, up to $5\times10^{10}$ $t_0$ simulation time | Reaction list consists of adding/removing hexagons. Study the growth and etching processes on Pt(111) | Ref.[15] |

1 Plane-Wave Density Functional Theory.
2 Bayesian Error Estimation Functional with van der Waals.
3 Perdew-Burke-Ernzerhof functional with D3 dispersion correction.
4 Nudged Elastic Band.
5 Perdew-Burke-Ernzerhof with D2 dispersion correction.
6 Mean-field approximation.

## References


1. Li, P.; Li, Z.; Yang, J. Dominant Kinetic Pathways of Graphene Growth in Chemical Vapor Deposition: The Role of Hydrogen. *The Journal of Physical Chemistry C* **2017**, *121*, 25949–25955. https://doi.org/10.1021/acs.jpcc.7b09622.
2. Evans, M.G.; Polanyi, M. Inertia and driving force of chemical reactions. *Trans. Faraday Soc.* **1938**, *34*, 11–24. https://doi.org/10.1039/TF9383400011.
3. Bell, R.P.; Hinshelwood, C.N. The theory of reactions involving proton transfers. *Proceedings of the Royal Society of London. Series A - Mathematical and Physical Sciences* **1936**, *154*, 414–429. Publisher: Royal Society, https://doi.org/10.1098/rspa.1936.0060.
4. Anglada, J.M.; Besalú, E.; Bofill, J.M.; Crehuet, R. Prediction of approximate transition states by Bell–Evans–Polanyi principle: I. *Journal of Computational Chemistry* **1999**, *20*, 1112–1129. _eprint: https://onlinelibrary.wiley.com/doi/pdf/10.1002/%28SICI%291096-987X%28199908%2920%3A11%3C1112%3A%3AAID-JCC2%3E3.0.CO%3B2-2, https://doi.org/10.1002/(SICI)1096-987X(199908)20:11<1112::AID-JCC2>3.0.CO;2-2.
5. Anand, M.; Nørskov, J.K. Scaling Relations in Homogeneous Catalysis: Analyzing the Buchwald–Hartwig Amination Reaction. *ACS Catal.* **2020**, *10*, 336–345. Publisher: American Chemical Society, https://doi.org/10.1021/acscatal.9b04323.
6. Wang, S.; Vorotnikov, V.; Sutton, J.E.; Vlachos, D.G. Brønsted–Evans–Polanyi and Transition State Scaling Relations of Furan Derivatives on Pd(111) and Their Relation to Those of Small Molecules. *ACS Catal.* **2014**, *4*, 604–612. https://doi.org/10.1021/cs400942u.
7. Falsig, H.; Shen, J.; Khan, T.S.; Guo, W.; Jones, G.; Dahl, S.; Bligaard, T. On the Structure Sensitivity of Direct NO Decomposition over Low-Index Transition Metal Facets. *Top Catal* **2014**, *57*, 80–88. https://doi.org/10.1007/s11244-013-0164-5.
8. Nørskov, J.K.; Bligaard, T.; Hvolbæk, B.; Abild-Pedersen, F.; Chorkendorff, I.; Christensen, C.H. The nature of the active site in heterogeneous metal catalysis. *Chem. Soc. Rev.* **2008**, *37*, 2163–2171. https://doi.org/10.1039/B800260F.
9. Garcia-Pintos, D.; Voss, J.; Jensen, A.D.; Studt, F. Hydrodeoxygenation of Phenol to Benzene and Cyclohexane on Rh(111) and Rh(211) Surfaces: Insights from Density Functional Theory. *J. Phys. Chem. C* **2016**, *120*, 18529–18537. https://doi.org/10.1021/acs.jpcc.6b02970.
10. Wang, S.; Petzold, V.; Tripkovic, V.; Kleis, J.; Howalt, J.G.; Skúlason, E.; Fernández, E.M.; Hvolbæk, B.; Jones, G.; Toftelund, A.; et al. Universal transition state scaling relations for (de)hydrogenation over transition metals. *Phys. Chem. Chem. Phys.* **2011**, *13*, 20760–20765. https://doi.org/10.1039/C1CP20547A.
11. Chen, S.; Gao, J.; Srinivasan, B.M.; Zhang, G.; Sorkin, V.; Hariharaputran, R.; Zhang, Y.W. An all-atom kinetic Monte Carlo model for chemical vapor deposition growth of graphene on Cu(1 1 1) substrate. *Journal of Physics: Condensed Matter* **2020**, *32*, 155401. https://doi.org/10.1088/1361-648x/ab62bf.
12. Hoffmann, M.J.; Bligaard, T. A Lattice Kinetic Monte Carlo Solver for First-Principles Microkinetic Trend Studies. *Journal of Chemical Theory and Computation* **2018**, *14*, 1583–1593, [https://doi.org/10.1021/acs.jctc.7b00683]. PMID: 29357239, https://doi.org/10.1021/acs.jctc.7b00683.
13. NASCAM (Nanoscale Modeling) @ONLINE. https://www.unamur.be/sciences/physique/ur/larn/logiciels/nascam.
14. Gaillard, P.; Chanier, T.; Henrard, L.; Moskovkin, P.; Lucas, S. Multiscale simulations of the early stages of the growth of graphene on copper. *Surface Science* **2015**, *637-638*, 11–18. https://doi.org/https://doi.org/10.1016/j.susc.2015.02.014.
15. Kong, X.; Zhuang, J.; Zhu, L.; Ding, F. The complementary graphene growth and etching revealed by large-scale kinetic Monte Carlo simulation. *npj Computational Materials* **2021**, *7*, 14. https://doi.org/10.1038/s41524-020-00489-y.
